# Electrochemical Synthesis of High‐Valent Metal Oxides via In Situ Oxidant Generation with Real‐Time Phase and Composition Control

**DOI:** 10.1002/advs.202514162

**Published:** 2025-09-23

**Authors:** Minjeong Kim, Dongwon Kim, Dongho Seo, Joon Yong Park, Ahyeon Ma, Yong‐Il Kim, Ki Min Nam

**Affiliations:** ^1^ Department of Chemistry and Institute for Future Earth Pusan National University Geumjeong‐gu Busan 46241 Republic of Korea; ^2^ Korea Research Institute of Standards and Science (KRISS) 267 Gajeong, Yuseong Daejeon 34113 Republic of Korea

**Keywords:** chloride oxidation, cobalt oxyhydroxide, seawater splitting, synthetic solid‐materials electrochemistry, tailored oxidants

## Abstract

Unlike conventional electrosynthesis, which primarily targets homogeneous reactions, this study introduces a scalable electrochemical strategy for synthesizing high‐valent metal oxide powders with controlled crystalline phases. Reactive oxidants are generated in situ and modulated by applied potential and electrolyte composition, enabling selective transformation of α‐Co(OH)_2_ into trigonal γ‐CoOOH or brucite‐like β‐CoOOH through oxidant‐mediated phase control. Metal ions such as Ir, Cu, or Mo are directly incorporated during the phase transformation, yielding doped γ‐CoOOH with tailored composition. The resulting γ‐CoOOH exhibits superior oxygen evolution reaction activity in alkaline media, with further enhancement achieved through metal doping. This approach is also applicable to manganese‐based systems, enabling selective synthesis of γ‐MnO_2_ and δ‐MnO_2_. The synthesis is readily integrated into a continuous‐flow system, allowing safe, waste‐minimized, and scalable production. These findings establish a new electrochemical methodology that unifies phase‐selective synthesis and in situ compositional control, offering a transformative route to the design of advanced electrocatalysts.

## Graphical abstract

This study presents a scalable electrochemical method for synthesizing high‐valent metal oxide powders with controlled phases and in situ metal doping. Reactive oxidants are generated and modulated electrochemically, enabling selective phase transformation and compositional tuning. The process is compatible with continuous‐flow systems, providing a safe, efficient, and general strategy for designing advanced electrocatalysts with enhanced performance.

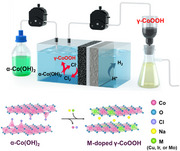

## 1 Introduction

The electrochemical synthesis method holds significant advantages for the continuous production of materials through either direct or mediated electron transfer processes.^[^ ^1^, ^2^, ^3^ ^]^ For example, the commercialized Chlor‐alkali process is well systematized and has been used with great success in the chemical industry.^[^ ^4^, ^5^, ^6^ ^]^ Additionally, electro‐organic synthesis has recently attracted significant research interest and has yielded intriguing results.^[^ ^7^, ^8^, ^9^, ^10^, ^11^ ^]^ However, despite these advances, electrochemical strategies for synthesizing heterogeneous powdered materials remain largely underexplored. Extending electrochemical synthesis to powdered materials enables precise control over crystalline structures and oxidation states, opening new opportunities to engineer material properties for catalytic applications.

Cobalt‐based hydroxides and oxyhydroxides, such as Co(OH)_2_ and CoOOH, have been widely studied as oxygen evolution reaction (OER) electrocatalysts due to their favorable redox properties, structural tunability, and intrinsic catalytic activity.^[^ ^12^, ^13^, ^14^ ^]^ Their performance is highly dependent on both the crystalline phase and dopant incorporation, making precise control over phase formation and composition a key synthetic challenge. Co(OH)_2_ exists in two polymorphs, α‐Co(OH)_2_ and β‐Co(OH)_2_ that differ in layer spacing and ion exchange behavior.^[^ ^15^, ^16^, ^17^, ^18^, ^19^, ^20^ ^]^ Upon oxidation, these hydroxides transform into CoOOH or Co_3_O_4_, with CoOOH being particularly active for the OER.^[^ ^21^ ^]^ CoOOH also exists in two major crystalline forms: β‐CoOOH, which possesses a rhombohedral structure with stable oxygen packing, and γ‐CoOOH, which adopts a trigonal framework with mixed‐valent cobalt (Co^3+^/Co^4+^) and intercalated cations.^[^ ^22^, ^23^ ^]^ The γ‐CoOOH phase exhibits superior OER activity due to its enhanced electronic conductivity and higher oxidation state.^[^ ^24^, ^25^, ^26^ ^]^ However, selective synthesis of γ‐CoOOH remains challenging, as it typically requires excess amounts of strong chemical oxidants (e.g., NaOCl or H_2_O_2_) and must proceed from an α‐Co(OH)_2_ phase precursor.^[^ ^27^, ^28^, ^29^ ^]^ The reliance on such oxidants raises serious safety and environmental concerns and imposes significant limitations on large‐scale or continuous synthesis. In situ generation of chemical oxidants and their immediate utilization for oxidative transformation offers a promising pathway to overcome these challenges, minimizing oxidant waste while enhancing safety. In addition to optimizing the crystal structure, compositional modification through doping has been demonstrated to further enhance the OER catalytic properties of CoOOH.^[^ ^30^, ^31^, ^32^, ^33^, ^34^, ^35^, ^36^, ^37^, ^38^, ^39^ ^]^ However, conventional doping strategies generally involve multistep post‐synthetic treatments, which limit structural uniformity and practical scalability. A facile doping strategy naturally integrated with phase‐selective synthesis would constitute a significant advance and define a new paradigm in synthetic methodology.

In this study, we present a controllable electrochemical synthesis method that enables phase‐selective transformation and simultaneous in situ metal‐ion doping of high‐valent metal oxides. By tuning the anodic potential and electrolyte composition, α‐Co(OH)_2_ is selectively transformed into either γ‐CoOOH or β‐CoOOH while allowing direct incorporation of dopant ions during the phase transformation. This single‐step process eliminates the need for additional post‐treatment steps, enabling the direct synthesis of structurally and compositionally tailored catalysts. This strategy is further applicable to the controlled synthesis of other high‐valent metal oxides, such as MnO_2_, allowing access to ramsdellite (γ‐MnO_2_) and birnessite (δ‐MnO_2_) phases. Importantly, adaptation to a continuous‐flow configuration allows for scalable, efficient, and waste‐minimized production of crystalline catalyst powders. Evaluation of the synthesized cobalt oxyhydroxides under alkaline OER conditions confirmed the superior performance of γ‐CoOOH, with further enhancements achieved through Cu, Ir, or Mo doping. These results establish this electrochemical synthesis as a general and versatile strategy for designing advanced electrocatalysts with simultaneous control over phase and composition.

## 2 Results and Discussion

### 2.1 Electrochemical In Situ Generation of Tailored Oxidants for the Phase‐Selective Synthesis of Cobalt Oxyhydroxides

A dimensionally stable anode, consisting of Ir and Ru coatings on a Ti plate (Ti/IrRu), was used for the electrochemical generation of the oxidants (Cl_2_, HOCl, O_2_) in 0.5 M NaCl (Equations (1), (2), (3)). The selective oxidation of α‐Co(OH)_2_ to the respective cobalt oxyhydroxides, β‐CoOOH and γ‐CoOOH, depends on the particular oxidant that is generated, while H_2_ is produced at the cathode (Figure S1, Supporting Information).

(1) $$2H_{2}O\to O_{2}+4H^{+}+4e^{-},\;E^{\circ }=1.23\;V$$

(2) $$2Cl^{-}\to Cl_{2}+2e^{-},\;\;E^{\circ }=1.36\;V$$

(3) $$Cl_{2}+H_{2}O\to \mathrm{HOCl}+H^{+}+Cl^{-}$$

(4) $$2\mathrm{Co}{\left(\mathrm{OH}\right)}_{2}+Cl_{2}\to 2\mathrm{CoOOH}\;+2Cl^{-}+2H^{+}$$

(5) $$\mathrm{Mn}{\left(\mathrm{OH}\right)}_{2}+O_{2}\to \mathrm{Mn}O_{2}+2OH^{-}$$

**Figure 1:**
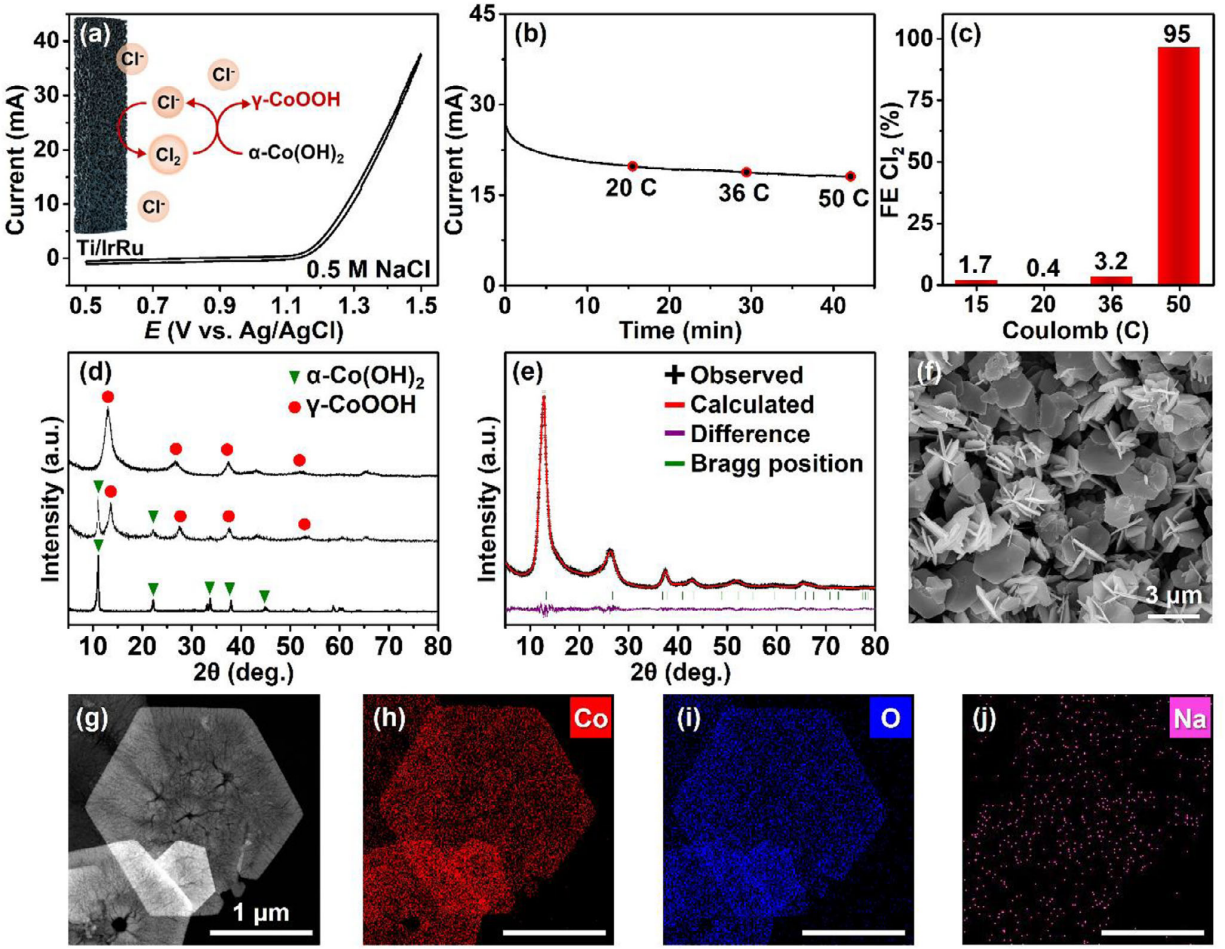
Selective electrochemical synthesis of γ‐CoOOH microplates via in situ oxidant generation. a) CV at a scan rate of 10 mV s^−1^ using a Ti/IrRu anode in 0.5 m NaCl with the presence of α‐Co(OH)_2_ microplates. b) Chronoamperometry measurement and c) FE of Cl_2_ relative to the applied charge at 1.4 V versus Hg/HgO in 0.5 m NaCl with α‐Co(OH)_2_ microplates. d) Variation in the XRD patterns during the conversion of α‐Co(OH)_2_ to γ‐CoOOH. e) Rietveld refinement analysis, and f) SEM image of γ‐CoOOH microplates. g) TEM image and STEM‐EDX elemental maps of h) Co, i) O, and j) Na.

The chloride oxidation reaction is the dominant oxidation reaction on the Ti/IrRu anode in 0.5 M NaCl (**Figure** **1**a; Figure S2, Supporting Information). This reaction selectively produces Cl_2_, which transforms into hypochlorous acid (HOCl, Equations 2 and 3) at pH levels below 7.^[^ ^40^, ^41^, ^42^, ^43^ ^]^ Cl_2_ is continuously generated using the chronoamperometric method on the Ti/IrRu anode with an approximate Faradaic efficiency (FE) of 95% (Figure 1b; Figure S2, Supporting Information).

Upon the addition of the α‐Co(OH)_2_ powder (Figure S3, Supporting Information) to the anode chamber, the generated Cl_2_ was immediately consumed (in real time) for the oxidation of α‐Co(OH)_2_ to γ‐CoOOH (Equation 4; Figure S1b, Supporting Information). Interestingly, at an applied charge below 36 C, which corresponds to the oxidative equivalent of the added α‐Co(OH)_2_, Cl_2_ was completely consumed and undetectable in the solution during the oxidation to γ‐CoOOH (Figure 1c). The added α‐Co(OH)_2_ crystalline particles were fully oxidized to γ‐CoOOH crystalline particles. When an additional charge was applied after complete oxidation to γ‐CoOOH, the Cl_2_ was detected with a FE of ≈95% (Figure 1c). The chemical oxidation of α‐Co(OH)_2_ to γ‐CoOOH, distinctly mediated by Cl_2_, quantitatively progressed through the repeated in situ generation and consumption of Cl_2_/HOCl as the oxidizing agent, thereby ensuring none of the oxidant was wasted during the synthesis. The progression of this oxidation reaction is evident from the X‐ray diffraction (XRD) patterns in Figure 1d.

Rietveld refinement analysis provided detailed structural information on γ‐CoOOH (Figure 1e). The diffraction peaks corresponded to the trigonal phase (Tables S1 and S2, Supporting Information), with lattice parameters as follows: space group, *R* 3 m (No. 160) and *Z* = 3; *a* (= *b*) = 0.2834(2) nm and *c* = 1.995(2) nm (Table S2, Supporting Information). The *R* factors are as follows: *R*_wp_ = 7.35%, *R*_p_ = 5.20%, *R*_e_ = 2.14%, GOF = 1.24. The result corresponded to the formula [H_0.54_Na_0.10_CoO_2_•0.36H_2_O]. The obtained γ‐CoOOH microplates retained the size and morphology of the α‐Co(OH)_2_ microplates during the oxidative phase transformation (Figure 1f). The rapid weight loss of nearly 17.8% at 250 °C on the thermogravimetric analysis (TGA) curve of γ‐CoOOH corresponds to dehydration and the phase transformation to Co_3_O_4_ with the loss of H_2_O (Figure S4a, Supporting Information).^[^ ^28^ ^]^ According to elemental mapping, Co and O atoms were evenly distributed throughout the microplate, with intercalated Na^+^ ions identified as well (Figure 1g–j). Energy dispersive X‐ray spectroscopy (EDX) revealed the presence of ≈1.6 wt.% of Na, indicating that the Na^+^ ions were intercalated within the γ‐CoOOH interlayers (Figure S5, Supporting Information). Interestingly, the oxidative phase transformation was accompanied by ion exchange: the intercalated Cl^−^ ions in the α‐Co(OH)_2_ were completely replaced by Na^+^ ions in the γ‐CoOOH layers (Figure S5, Supporting Information). The use of electrolytes containing other metal salts such as KCl and CsCl similarly resulted in the selective intercalation of the corresponding cations (K^+^, Cs^+^) into the layered structure to form the γ‐CoOOH phase (Figures S5 and S6, Supporting Information). To evaluate the detailed oxidation states, X‐ray photoelectron spectroscopy (XPS) was performed, confirming that γ‐CoOOH predominantly exhibits a Co^3+^ oxidation state, while the intercalated Na, K, and Cs ions are present in the +1 oxidation state (Figure S7, Supporting Information).

The OER is a dominant reaction on Ti/IrRu electrode under high pH conditions (0.5 m NaCl and 2 m NaOH).^[^ ^44^, ^45^ ^]^ O_2_ was selectively generated using the chronoamperometric method (Figure S8, Supporting Information). The selective oxidation of α‐Co(OH)_2_ to β‐CoOOH occurred by generating O_2_ under alkaline conditions (Figure S1c, Supporting Information), and rapid chemical oxidation resulted in a complete transformation to the brucite‐like phase of β‐CoOOH (Figure S8d, Supporting Information). Unlike for γ‐CoOOH, intercalated cations were not detected in β‐CoOOH. The lattice parameters derived from the Rietveld refinement analysis are as follows: *P* $\;\bar{3}$ m 1 (No. 164); *a* (= *b*) = 0.2863(4) nm and *c* = 1.309(2) nm (Table S1, Supporting Information). The scanning electron microscopy (SEM) image revealed that the size and morphology of the α‐Co(OH)_2_ microplates during the oxidative phase transformation to β‐CoOOH microplates were similar to those of α‐Co(OH)_2_ microplates (Figure S9, Supporting Information). However, the surface became rough, and nanoplates formed on the microplate during the phase transformation process under basic conditions (Figure S9d, Supporting Information). According to elemental mapping, Co and O atoms were homogeneously distributed throughout the microplate, with no intercalated ions present (Figure S10, Supporting Information). The consecutive weight loss (about 16%) observed from the TGA curve corresponded to dehydration and the phase transition to Co_3_O_4_ (Figure S4b, Supporting Information). This weight loss occurred more slowly compared to that of γ‐CoOOH, and corresponds to the higher thermal stability of β‐CoOOH.

**Figure 2:**
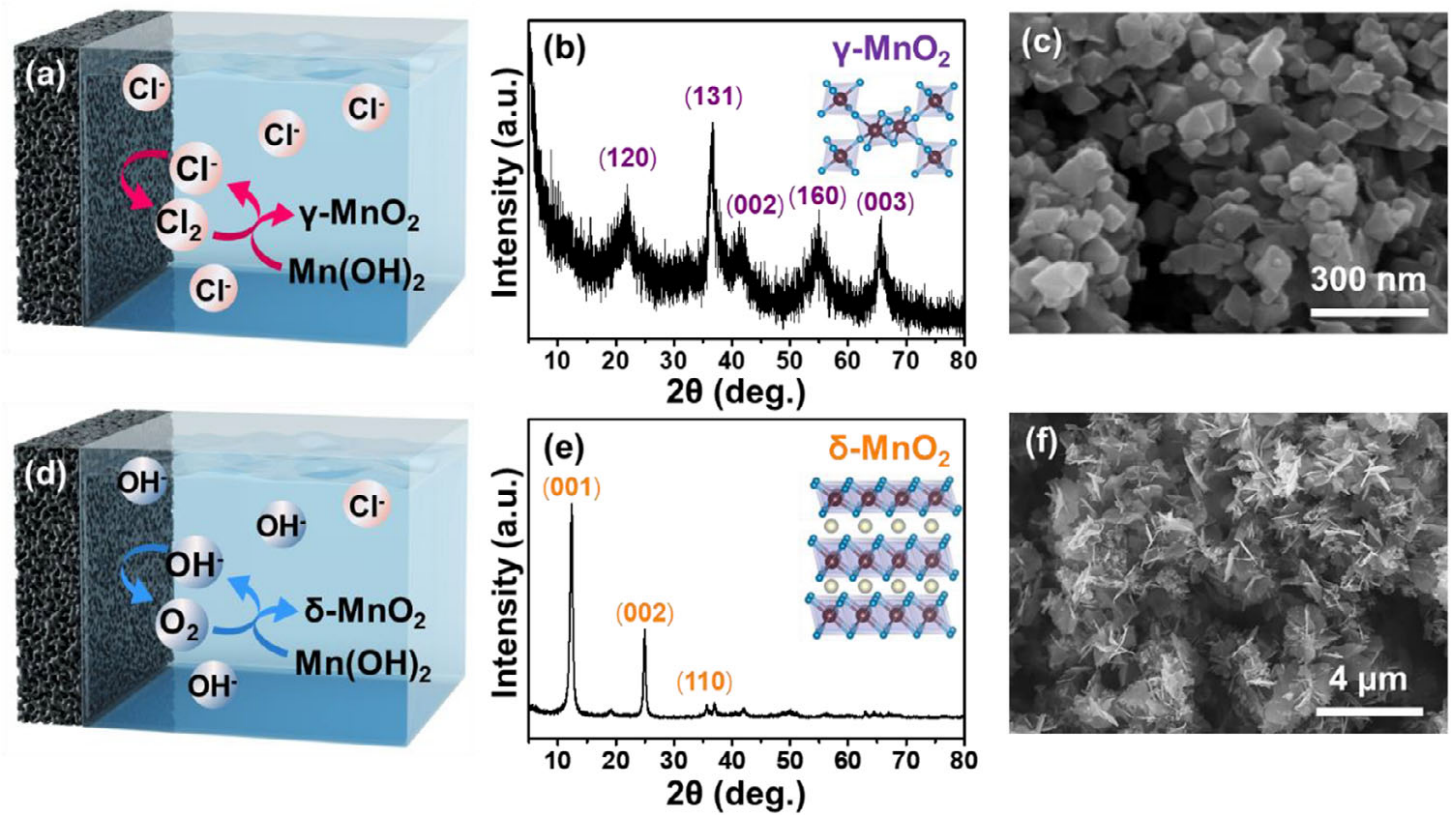
Selective electrochemical synthesis of MnO_2_ polymorphs via in situ oxidant generation. a) Schematic representation of in situ Cl_2_ generation in 0.5 m NaCl for the oxidative conversion of Mn(OH)_2_ to γ‐MnO_2_ nanoparticles. b) XRD pattern and c) SEM image of γ‐MnO_2_ nanoparticles. d) Schematic representation of in situ O_2_ generation in 0.5 m NaCl and 2 m NaOH for the oxidative conversion of Mn(OH)_2_ to δ‐MnO_2_ nanosheets. e) XRD pattern and f) SEM image of δ‐MnO_2_ nanosheets.

The broader applicability of this synthesis method was investigated by using it for the phase‐selective synthesis of MnO_2_. Mn(OH)_2_ nanoplates were prepared via a simple precipitation method (Figure S11, Supporting Information), whereupon they were selectively oxidized to MnO_2_ phases (Equation 5), depending on the generated oxidants (**Figure** **2**). The generated Cl_2_ was consumed in the oxidation of Mn(OH)_2_ to MnO_2_ (Figure 2a), with the resulting MnO_2_ diffraction peaks corresponding to those of the ramsdellite structure (γ‐MnO_2_) (Figure 2b). The γ‐MnO_2_ is an intergrowth structure composed of ramsdellite and rutile domains, where the relative fraction of these domains can slightly influence the peak positions.^[^ ^46^ ^]^ The SEM image of the γ‐MnO_2_ nanoparticles revealed that the morphology had changed from that of the Mn(OH)_2_ nanoplates as a result of the oxidative phase transformation (Figure 2c). On the other hand, Mn(OH)_2_ was selectively oxidized by O_2_ generated under basic conditions (Figure 2d), with fast chemical oxidation resulting in oxidative transformation to the birnessite phase of MnO_2_ (δ‐MnO_2_, Figure 2e). In this case, the morphology changed completely from that of the Mn(OH)_2_ nanoplates to the interconnected nanosheets of δ‐MnO_2_ (Figure 2f). Interestingly, the oxidative phase transformation causes Na^+^ ions to intercalate between the δ‐MnO_2_ layers (Figure S12, Supporting Information). The use of other electrolytes such as KCl and CsCl solutions similarly resulted in the intercalation of the cations (K^+^, Cs^+^) into the layered structure to form the δ‐MnO_2_ phase (Figure S13, Supporting Information). The δ‐MnO_2_ adopts a birnessite‐type layered structure, and the interlayer spacing is highly sensitive to the type of intercalated cations. Since hydrated Na^+^ ions are slightly larger than hydrated K^+^ ions, a small shift in the (001) diffraction peaks can occur depending on the dominant species present between the layers (Figure S14, Supporting Information). To evaluate the detailed oxidation states, XPS was performed, confirming that δ‐MnO_2_ predominantly exhibits a Mn^4+^ oxidation state, while the intercalated Na, K, and Cs ions are present in the +1 oxidation state (Figure S15, Supporting Information). These results indicate the broad applicability of this electrochemical synthesis approach for the phase‐selective preparation of crystalline high‐valent metal oxides.

### 2.2 Consecutive Synthesis of High‐Valent Metal Oxides using an Electrochemical Flow System

**Figure 3:**
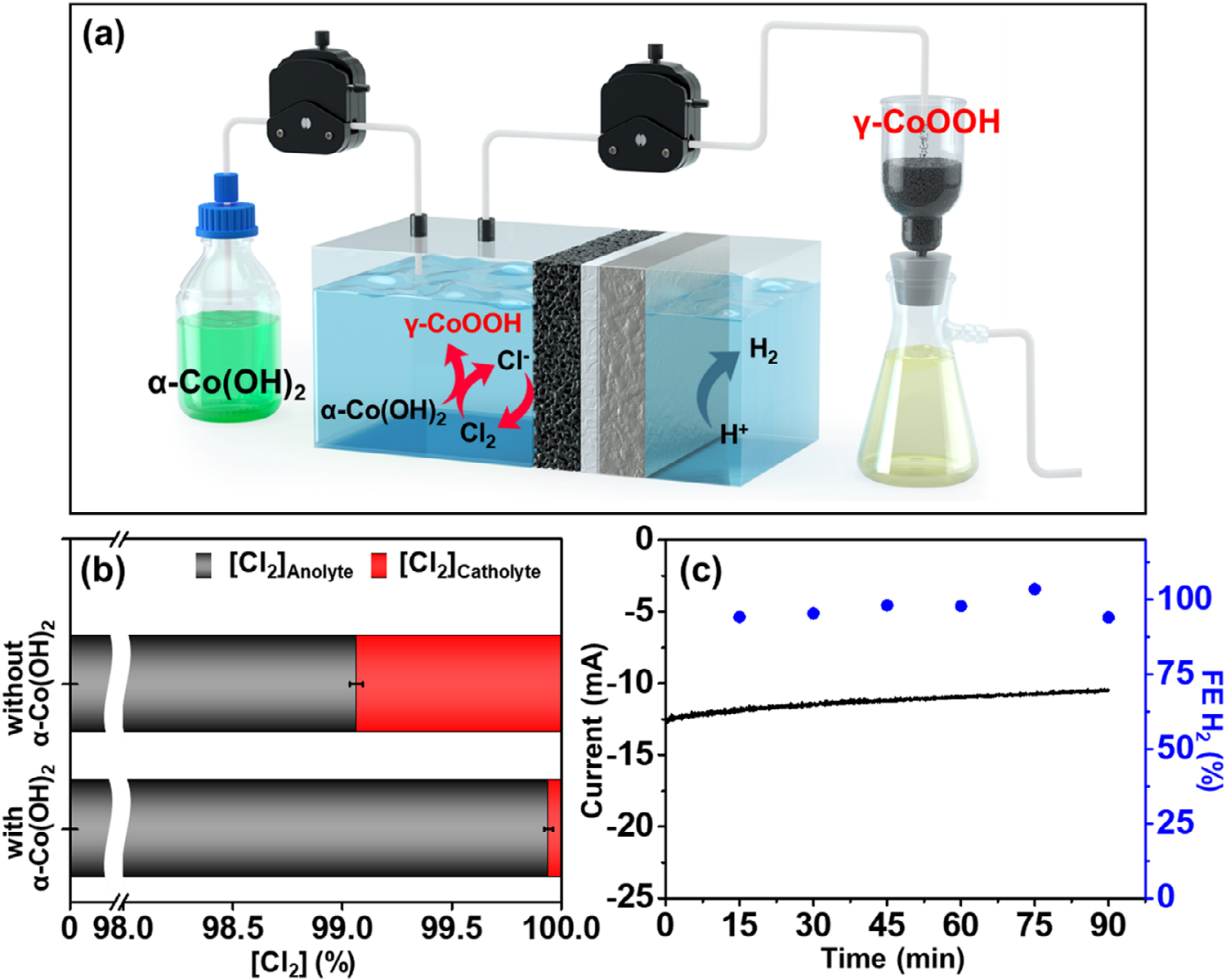
Continuous electrochemical synthesis of γ‐CoOOH and its effect on product selectivity. a) Schematic synthesis diagram of γ‐CoOOH microplates in 0.5 m NaCl. b) Comparison of Cl_2_ crossover ratio with and without α‐Co(OH)_2_ at a flow rate of 0.5 sccm. c) FE of H_2_ at the cathode in 0.5 m NaCl.

To demonstrate the scalability of this electrochemical synthesis method, γ‐CoOOH was consecutively synthesized using a Ti/IrRu electrode in a flow cell system. Cl_2_ was generated in situ via chronoamperometry, while a peristaltic pump continuously supplied dispersed α‐Co(OH)_2_ powder in 0.5 m NaCl to the anode compartment (**Figure** **3**a). The α‐Co(OH)_2_ was immediately transformed into γ‐CoOOH, and the γ‐CoOOH microplates were obtained using a simple filtration process (Figure S16, Supporting Information). The flow rate was adjustable from 0.5 to 2.5 sccm, and by tuning the applied potential, the Cl_2_ generation rate could be precisely controlled, thereby enabling regulation of the γ‐CoOOH synthesis rate to match the desired production level in the flow cell (Figure S17a, Supporting Information). The progress of the oxidative transformations was monitored by XRD analysis, which revealed the peaks corresponding to γ‐CoOOH (Figure S17b, Supporting Information). Note that membrane degradation and cathode deterioration can occur due to Cl_2_/HOCl generated during brine (or seawater) electrolysis, as these oxidants may permeate into the cathode chamber, reducing FE of H_2_.^[^ ^45^ ^]^ Even with high‐quality membranes, crossover of Cl_2_/HOCl has been observed, with ≈1% detected in the catholyte based on calibration curve analysis (Figure S18, Supporting Information). However, when α‐Co(OH)_2_ is introduced at the anode chamber, the generated Cl_2_/HOCl is immediately consumed, and only trace amounts are detected in the cathode chamber (Figure 3b).

Under these conditions, γ‐CoOOH is synthesized at the anode, while the Pt cathode enables H_2_ generation with a FE of ≈99% in 0.5 m NaCl, demonstrating that this approach not only enables selective material synthesis but also significantly enhances membrane durability and H_2_ production efficiency (Figure 3c). To enhance synthetic convenience, seawater was used instead of 0.5 m NaCl, and the resulting γ‐CoOOH exhibited an identical crystal structure. However, SEM‐EDX analysis revealed the presence of small amounts of Mg^2+^, Ca^2+^, and K^+^ co‐intercalated with Na^+^ (Figure S19, Supporting Information). Since all these ions exist in intercalated forms, they are exchanged with K^+^ under OER conditions in 1 m KOH, as confirmed by post‐reaction analysis. Therefore, seawater can be employed as a convenient alternative to NaCl for synthesizing γ‐CoOOH catalysts without compromising their structural integrity or catalytic performance.

Continuous production of H_2_ requires the efficient consumption of toxic Cl_2_ in seawater splitting, a challenge fully addressed by this synthesis method. Compared to chemical synthesis, which requires the addition of external oxidants, the electrochemical flow system enables continuous operation, which makes it suitable for large‐scale synthesis with in situ generated oxidants. Because the distinctly mediated oxidation to γ‐CoOOH proceeds quantitatively through the in situ generation of Cl_2_, none of the oxidizing agent is wasted during the Cl_2_‐mediated electrochemical process. Similarly, α‐Co(OH)_2_ could be selectively oxidized to β‐CoOOH through a chemical oxidation process driven by O_2_ evolution under basic conditions using a continuous flow system (Figure S20, Supporting Information). Rapid oxidation led to the complete phase transformation into β‐CoOOH. Furthermore, the same flow system can be applied for the continuous synthesis of δ‐MnO_2_. These results highlight the versatility of the flow‐based synthesis platform for scalable and phase‐controlled production of high‐valent transition metal oxides.

### 2.3 Electrocatalytic Reaction on the Cobalt Oxyhydroxides

**Figure 4:**
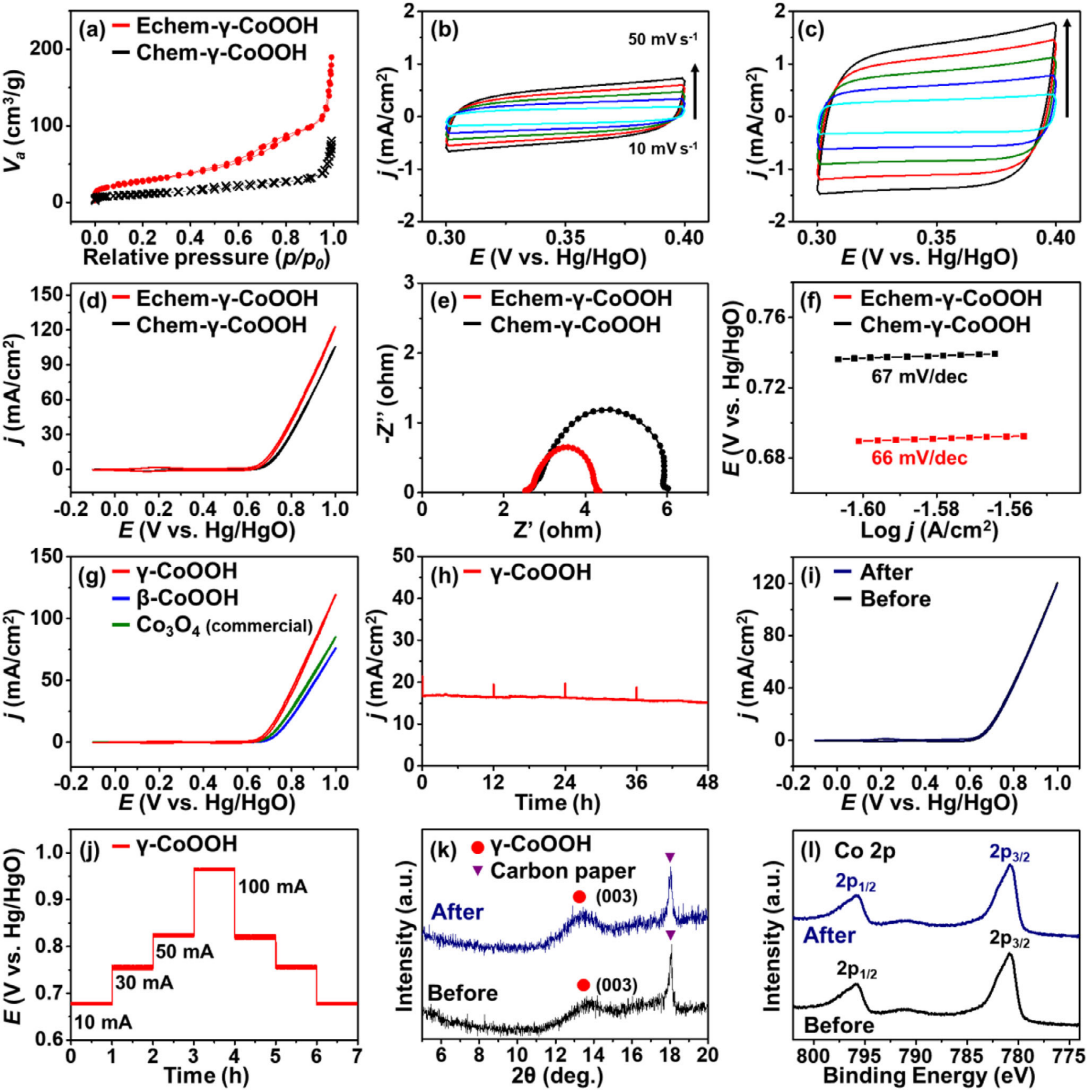
Electrochemical activity and stability comparison of chemically and electrochemically synthesized γ‐CoOOH. a) N_2_ adsorption/desorption isotherms of Chem‐γ‐CoOOH, and Echem‐γ‐CoOOH. CVs of b) Chem‐γ‐CoOOH, and c) Echem‐γ‐CoOOH at different scan rates. d) CVs, e) Nyquist plots at 0.70 V versus Hg/HgO, and f) Tafel slopes of Chem‐γ‐CoOOH, and Echem‐γ‐CoOOH in 1 m KOH. g) CVs of Echem‐γ‐CoOOH, Echem‐β‐CoOOH, and commercial Co_3_O_4_ in 1 m KOH (scan rate: 10 mV s^−1^). h) Chronoamperometry measurement of γ‐CoOOH at 0.75 V versus Hg/HgO for 48 h in 1 m KOH. i) CVs of γ‐CoOOH before and after the stability test. j) Dependence of the chronopotentiometry measurement of γ‐CoOOH on the applied potentials in 1 m KOH. k) XRD and l) XPS analyses of γ‐CoOOH before and after the stability test.

To evaluate their catalytic performance, the synthesized high‐valent metal oxides were tested in alkaline water electrolysis. Given the relatively low OER activity of the MnO_2_ phases, this section focuses on electrochemically prepared γ‐CoOOH and β‐CoOOH as active electrocatalysts. The physicochemical properties of the electrochemically synthesized γ‐CoOOH (Echem‐γ‐CoOOH) were compared with those of its chemically synthesized counterpart (Chem‐γ‐CoOOH). They were chemically or electrochemically synthesized from the same α‐Co(OH)_2_ powder. Notably, Brunauer–Emmett–Teller (BET) measurements showed that the Echem‐γ‐CoOOH had a larger surface area than the Chem‐γ‐CoOOH (**Figure** **4**a; Figure S21, Supporting Information). While the electrochemically active surface area (ECSA) does not provide an absolute measure, it serves as a useful proxy for comparing relative surface areas in metal oxide samples. ECSA analysis revealed a higher value for the Echem‐γ‐CoOOH, supporting its relatively larger surface area (Figure 4b,c).

These results are in good agreement with the peak broadening observed on the XRD results (Figure S22, Supporting Information). The larger surface area of Echem‐γ‐CoOOH is attributed to the rapid oxidation of α‐Co(OH)_2_ by in situ generated Cl_2_ under mildly acidic conditions. Cl_2_, which has a higher standard electrode potential than NaOCl used in the chemical synthesis of Chem‐γ‐CoOOH, promotes faster nucleation and phase transformation. This rapid reaction likely suppresses particle growth and leads to the formation of smaller microstructures, resulting in a higher surface area for the electrochemically synthesized γ‐CoOOH. Notably, this electrochemical synthesis method allows for increased surface area, which can be beneficial for enhancing its catalytic performance.

Cyclic voltammetry (CV) was used to study the electrochemical OER in 1 m KOH solution of Echem‐γ‐CoOOH in comparison with that of Chem‐γ‐CoOOH. Echem‐γ‐CoOOH exhibited superior OER activity compared to its chemically synthesized counterpart due to its enhanced surface area (Figure 4d). Electrochemical impedance spectroscopy (EIS) revealed total resistances of 6.0 Ω for Chem‐γ‐CoOOH, and 4.3 Ω for Echem‐γ‐CoOOH (Figure 4e), with the fast charge transfer corresponding to Echem‐γ‐CoOOH, aligning well with its superior OER activity. Although the Tafel slopes of Echem‐γ‐CoOOH (66 mV dec^−1^) and Chem‐γ‐CoOOH (67 mV dec^−1^) were nearly identical (Figure 4f), the onset potential of Echem‐γ‐CoOOH was slightly more negative, suggesting that the increased surface area may facilitate earlier activation of the OER. These results collectively support the enhanced suitability of Echem‐γ‐CoOOH for electrochemical OER under alkaline conditions. This highlights the effectiveness of this synthesis method for producing an efficient OER catalyst, with the ability to control both the crystal structure and surface area.

The OER performance of Echem‐γ‐CoOOH was additionally assessed in comparison to that of the Echem‐β‐CoOOH and representative commercial Co_3_O_4_ nanoparticles, a widely used electrocatalyst (Figure 4g). The γ‐CoOOH electrode exhibited superior OER activity to both commercial Co_3_O_4_ and β‐CoOOH. The EIS and Tafel slope results also indicated that γ‐CoOOH is a highly efficient catalyst for the electrochemical OER (Figure S23, Supporting Information). The FE of the OER on the samples was evaluated by measuring the released O_2_ using gas chromatography during the chronoamperometry experiment (Figure S24, Supporting Information). The FE of O_2_ generation on all samples was ≈100%, indicating that most of the current from the electrochemical measurements were consumed for O_2_ generation.

The stability was evaluated by conducting a chronoamperometry experiment at 0.75 V versus Hg/HgO in 1 m KOH for 48 h, with the electrolyte refreshed every 12 h (Figure 4h). After an initial decrease, the current of the γ‐CoOOH electrode stabilized at a steady‐state level and remained constant throughout the 48 h test. The CV measurements before and after the reaction were nearly identical, indicating excellent structural and electrochemical stability under alkaline conditions (Figure 4i). In addition, the γ‐CoOOH electrode exhibited stable OER performance across a wide range of current densities, including relatively high values (Figure 4j), demonstrating its robustness and potential for further development toward practical water electrolysis applications. The crystal structure and surface composition of γ‐CoOOH were systematically examined by XRD and XPS before and after the stability test to evaluate structural resilience during the OER. XRD patterns exhibited no noticeable changes in peak positions or intensities after electrolysis, confirming that the γ‐CoOOH maintained its crystalline integrity throughout the reaction (Figure 4k). XPS analysis further indicated that the cobalt oxidation states remained unchanged, as evidenced by the consistent Co 2p peak positions and intensities before and after OER test (Figure 4l; Figure S25, Supporting Information). SEM also showed that the microplate morphology of γ‐CoOOH was preserved after prolonged electrochemical operation (Figure S26, Supporting Information). These results collectively highlight the exceptional structural robustness, chemical stability, and morphological retention of γ‐CoOOH under alkaline conditions, indicating its potential as a durable anode catalyst for long‐term water electrolysis. In comparison, although the electrochemically synthesized β‐CoOOH displayed lower intrinsic OER activity, it also demonstrated remarkable structural and electrochemical stability under identical alkaline conditions (Figure S27, Supporting Information), suggesting its potential as a stable catalytic scaffold for further activity enhancement through compositional tuning or heteroatom doping.

### 2.4 Selective Metal Ion Doping into γ‐CoOOH Lattices for Improved Electrocatalytic Reaction

**Figure 5:**
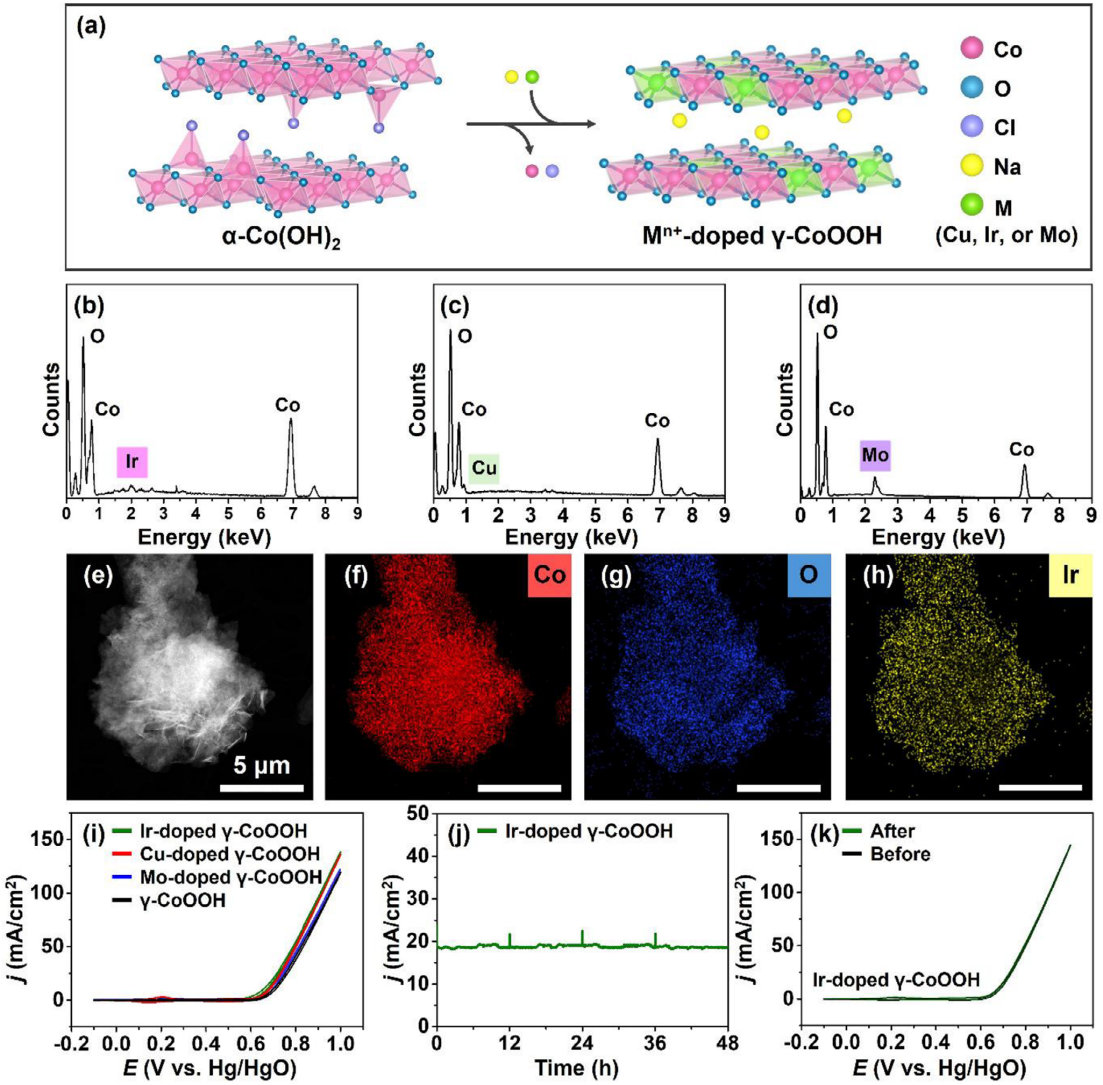
Enhanced electrocatalytic performance of doped γ‐CoOOH via electrochemical incorporation of metal ions. a) Schematic illustration of the electrochemical doping process. SEM‐EDX spectrum confirming the successful incorporation of b) Ir, c) Cu, and d) Mo, respectively into γ‐CoOOH. e) TEM image and STEM‐EDX elemental maps of f) Co, g) O, and h) Ir. i) CVs at a scan rate of 10 mV s^−1^ of Ir‐doped γ‐CoOOH, Cu‐doped γ‐CoOOH, Mo‐doped γ‐CoOOH, γ‐CoOOH in 1 m KOH. j) Chronoamperometric measurement of 0.70 versus Hg/HgO and k) CVs of Ir‐doped γ‐CoOOH before and after the chronoamperometric measurement.

Metal ions such as Ir^4+^ and Mo^6+^, when inserted into γ‐CoOOH, are known to positively influence the OER activity.^[^ ^30^, ^32^ ^]^ During the electrochemical synthesis process, when metal ions such as Ir^4+^, Mo^6+^, or Cu^2+^ are present in the electrolyte, spontaneous doping occurs concurrently with the oxidation of α‐Co(OH)_2_ to γ‐CoOOH (**Figure** **5**a). This represents a significant advantage of the electrochemical method, enabling simultaneous phase transformation and compositional tuning without requiring additional post‐synthetic steps. The resulting metal‐doped γ‐CoOOH retains the crystallinity and microplate morphology of the undoped γ‐CoOOH material, indicating that the doping process does not disrupt the structural integrity (Figure S28, Supporting Information). Moreover, intercalated Na^+^ ions, which are observed in γ‐CoOOH, are barely detectable in the doped samples, suggesting that charge compensation is instead achieved by the incorporated high‐valent metal ions. The doping levels are estimated to reach up to ≈1.2% for Ir^4+^, 6.3% for Cu^2+^, and 2.6% for Mo^6+^, with the values representing relative metal contents normalized to Co (Figure 5b–d; Figure S29, Supporting Information).

To accurately determine the amount of dopants, we performed inductively coupled plasma–optical emission spectroscopy (ICP‐OES) measurements for multiple samples, including γ‐CoOOH (Na^+^ intercalated), Cu‐doped γ‐CoOOH, and Ir‐doped γ‐CoOOH and re‐evaluated the metal contents accordingly. The elemental contents obtained from ICP‐OES were ≈6.2% for Na^+^, 1.2% for Ir^4+^, and 7.4% for Cu^2+^, with the values representing relative metal contents normalized to Co (Figure S30, Supporting Information). Despite minor differences between ICP‐OES and SEM‐EDS, the results show good consistency, confirming the reliability of both methods. Moreover, the extent of dopant incorporation can be readily controlled by adjusting the concentration of metal ions in the electrolyte. For example, Cu^2+^ was incorporated at levels of 1.8%, 3.8%, and 6.3%, depending on the Cu precursor concentration (Figure S31, Supporting Information), with ≈7% representing the saturation limit, beyond which no further increase was observed, even at higher precursor concentrations. Elemental mapping revealed that Co, O, and dopant (e.g., Ir) atoms were uniformly distributed across the microplate, indicating homogeneous doping within the γ‐CoOOH structure (Figure 5e–h).

CV analysis was conducted to evaluate the OER performance of metal‐doped γ‐CoOOH electrodes (Ir, Mo, or Cu) in 1 m KOH, compared to undoped γ‐CoOOH. All doped samples exhibited enhanced OER activity, with the Ir‐doped γ‐CoOOH showing the highest catalytic performance among them (Figure 5i). The Ir‐doped γ‐CoOOH electrode maintained a stable current density over 48 h, with the electrolyte refreshed every 12 h, indicating steady‐state catalytic behavior under alkaline conditions (Figure 5j). Furthermore, the CV profiles recorded before and after electrolysis were nearly identical, demonstrating the excellent electrochemical stability of the catalyst under alkaline conditions (Figure 5k). The enhanced OER activity of Ir‐doped γ‐CoOOH arises from two complementary effects.^[^ ^30^, ^31^, ^38^ ^]^ First, electronic modulation by Ir incorporation perturbs the γ‐CoOOH electronic structure (e.g., Co d‐band and O 2p‐band center), which optimizes the adsorption energies of OER intermediates and reduces the free‐energy barrier of the rate‐determining step.^[^ ^30^ ^]^ Second, interfacial stabilization occurs via the formation of Ir─OH─Co motifs, where steric and hydrogen–bonding interactions stabilize the *OOH intermediate while maintaining optimal *O binding, further accelerating OER kinetics.^[^ ^31^ ^]^

XPS analysis confirmed that Ir exists predominantly in the +4 oxidation state (Figure S32, Supporting Information) and is uniformly dispersed within the γ‐CoOOH microplates (Figure 5h), while XRD patterns showed no evidence of IrO_2_ particles, indicating atomic‐level incorporation of Ir into the γ‐CoOOH lattice. Consistently, when only the Ir precursor was used under the same synthesis conditions, no IrO_2_ particles were formed, further supporting the absence of segregated Ir‐based phases. Therefore, both the electronic modulation and interfacial stabilization mechanisms are likely to operate synergistically in our system, consistent with previous DFT‐supported studies.^[^ ^30^, ^31^ ^]^

XRD analysis revealed no significant changes in peak positions or intensities, confirming that the crystalline structure of Ir‐doped γ‐CoOOH was preserved during the OER. SEM imaging further confirmed the retention of the microplate morphology, while EDX analysis showed no detectable loss of Ir content, indicating that the dopant remained stably incorporated throughout the reaction (Figure S33, Supporting Information). Collectively, these results demonstrate the structural integrity, morphological robustness, and compositional stability of Ir‐doped γ‐CoOOH, supporting its potential as a durable and efficient OER catalyst for alkaline water electrolysis.

Notably, Cu‐doped γ‐CoOOH exhibited OER performance comparable to that of the Ir‐doped sample (Figure 5i), highlighting the effectiveness of transition metal doping during electrochemical synthesis. These findings demonstrate the capability of our method to produce structurally robust and catalytically enhanced γ‐CoOOH via in situ doping, motivating further investigation into dopant‐specific effects.

## 3 Conclusion

This work presents a versatile electrochemical platform for the synthesis of high‐valent metal oxides with phase and compositional control, enabled by the real‐time generation of reactive oxidants. The method enables selective phase transformation of α‐Co(OH)_2_ into either γ‐CoOOH using Cl_2_ or β‐CoOOH using O_2_. This selectivity is governed by the oxidant identity, which can be modulated by tuning the applied potential and electrolyte composition. The generality of this principle was validated by applying the same strategy to MnO_2_, successfully achieving phase‐selective synthesis of either γ‐MnO_2_ or δ‐MnO_2_ depending on the electrochemical conditions. These results demonstrate that the approach is not limited to cobalt systems but is extendable to other transition metal oxides as well. Furthermore, the platform is compatible with continuous‐flow systems, allowing for precise oxidant generation and the scalable production of high‐valent metal oxide powders while avoiding the hazards associated with bulk chemical oxidants. This design enhances safety, reduces chemical waste, and enables precise real‐time control over crystalline phase formation, which is difficult to achieve with conventional batch synthesis methods.

One of the key advantages of this approach is the ability to directly incorporate catalytically active metal ions such as Ir^4+^, Mo^6+^, or Cu^2+^ during the oxidation‐driven phase transformation of α‐Co(OH)_2_ to γ‐CoOOH. This in situ doping process eliminates the need for post‐synthetic modification while enabling precise compositional tuning. Structural and compositional analyses confirmed that the resulting doped γ‐CoOOH materials retained crystallinity and morphology comparable to the undoped sample. Importantly, the doped materials exhibited minimal Na⁺ intercalation, suggesting that the charge balance was compensated by the incorporated metal ions. The doping levels were tuned by varying the metal precursor concentration in the electrolyte, resulting in a homogeneous dopant distribution. All doped samples exhibited enhanced OER activity, with the Ir‐doped γ‐CoOOH showing the highest catalytic performance among them, and the dopant remaining stably incorporated throughout the reaction. The structural integrity, morphological robustness, and compositional stability of Ir‐doped γ‐CoOOH underscore its potential to serve as a durable and efficient OER catalyst for alkaline water electrolysis. Notably, Cu‐doped γ‐CoOOH exhibited OER activity comparable to Ir‐doped γ‐CoOOH, highlighting the potential of earth‐abundant, non‐noble metal dopants as cost‐effective alternatives. These findings demonstrate the broad applicability of this electrochemical platform for discovering and evaluating dopants that enhance electrocatalytic performance. Overall, this electrochemical synthesis strategy provides a unified platform for phase‐selective transformation, in situ doping, and scalable production of high‐valent metal oxide catalysts.

## Conflict of Interest

The authors declare no conflict of interest.

## Supporting information



Supporting Information

## Data Availability

The data that support the findings of this study are available from the corresponding author upon reasonable request.
