# Supplementary material for: Electrochemical Synthesis of High‐Valent Metal Oxides via In Situ Oxidant Generation with Real‐Time Phase and Composition Control
Source: Adv Sci (Weinh). 2025 Sep 23;12(46):e14162. doi: 10.1002/advs.202514162 (PMC12697890; doi:10.1002/advs.202514162)
Supplement: Supplementary file 1 — Supporting Information [file ADVS-12-e14162-s001.pdf]

Supporting Information

Electrochemical Synthesis of High-Valent Metal Oxides via In Situ Oxidant Generation with Real-Time Phase and Composition Control

*Minjeong Kim,[a] Dongwon Kim,[a] Dongho Seo,[a] Joon Yong Park,[a] Ahyeon Ma,[a] Yong-Il Kim,[b] and Ki Min Nam[a,]**

M. Kim, D. Kim, D. Seo, J. Y. Park, A. Ma, K. M. Nam
Department of Chemistry and Institute for Future Earth, Pusan National University, Geumjeong-gu, Busan 46241, Republic of Korea
E-mail: kimin.nam@pusan.ac.kr

Y.-I. Kim
Korea Research Institute of Standards and Science (KRISS), 267 Gajeong, Yuseong, Daejeon, 34113, Republic of Korea

Experimental Section

Materials. Cobalt (II) chloride hexahydrate (97%, Sigma-Aldrich), manganese (II) chloride tetrahydrate (98%, Daejung), sodium molybdate dihydrate (≥99%, Sigma-Aldrich), copper (II) chloride (97%, Sigma-Aldrich), iridium(IV) chloride hydrate (Sigma-Aldrich) used as received, were utilized as metal precursor salt. Hexamethylenetetramine (HMT, 99.0%, Sigma-Aldrich), lithium hydroxide monohydrate (99.95%, Sigma-Aldrich), lithium chloride (99%, Sigma-Aldrich), sodium hydroxide (97%, Daejung), sodium chloride (99.5%, Oriental Chemical Industry), potassium hydroxide (85%, Daejung), potassium chloride (99%, Daejung), cesium hydroxide hydrate (99.9%, Thermo Fisher Scientific), cesium chloride (99%, Daejung), sodium hypochlorite (9.0-11.0%, Daejung), cobalt (II, III) oxide (99.5%, Sigma-Aldrich) were used without further purification. Acetone (99.5%) and ethanol (99.5%) were sourced from

Daejung Chemicals. Dimensionally stable anodes (DSA (Ti/IrRu and Ti/IrTa, 1.8 cm^2, Techwin), glassy carbon (GC, 0.5 mm thick, Type G, HTW GmbH, Germany) were used as the electrode for the chloride oxidation reaction. Active chlorine reagents, R1 solution (potassium iodide, KI, < 20%) and R2 solution (acetic acid glacial, < 30%), were purchased from Shenzhen Sinche Technology Co., Ltd. Carbon paper (MGL 190, AvCarb) was used as a substrate for the electrochemical experiments.

Characterization of materials. Powder X-ray diffraction (PXRD) measurements (Rigaku SmartLab diffractometer, Cu Kα1, 40 kV, 30 mA) were conducted to identify the phase purity and crystal structure of cobalt hydroxide. Data were acquired under ambient conditions with a detector step size of 0.01° in the range of $5° \leq 2\theta \leq 80°$ with a primary monochromator Ge(111) in reflection mode. The electrodes were characterized by SEM (HR-SEM, Zeiss SUPRA 25), TEM (Talos F200X, operated at 200 kV.) and X-ray photoelectron spectroscopy (XPS) measurements by using Al *Kα* X-ray source. (Pass energy level: 40 eV). UV–Vis diffuse reflectance spectra were acquired using a UV-3600 UV-Vis-NIR spectrophotometer equipped with and the UV quartz cell for wavelengths ranging from 300 to 1500 nm with the references. Inductively coupled plasma optical emission spectrometry (ICP-OES, Avio 550, Perkin Elmer) measurements were performed after sample pretreatment with a microwave digestion system (Multiwave 7000, Anton Paar). The surface area and pores were analyzed by the Brunauer Emmett–Teller (BET) method (BELSORP–mini X).

Chemical synthesis of α-$Co(OH)_2$. α-$Co(OH)_2$ was synthesized using a chemical precipitation method. The precursor solution was prepared by dissolving $CoCl_2 \cdot 6H_2O$ (10 mM), NaCl (50 mM), and hexamethylenetetramine (HMT, 60 mM) in a mixture of ethanol and deionized water (1:9, 150 mL). The mixture was continuously stirred and heated at 95 °C for 1 hour, resulting in the formation of a green precipitate. The reaction temperature can be

adjusted to 80 °C or 85 °C for 2 hours to control the particle size (Supplementary Fig.3). The precipitate was collected and washed thoroughly with ethanol and deionized water to remove impurities. The product was then dried under vacuum at room temperature to obtain a green powder.

Electrochemical synthesis of γ-CoOOH (Echem-γ-CoOOH). Echem-γ-CoOOH was synthesized via electrochemical oxidation using chlorine-mediated oxidation. The electrochemical cell consisted of the commercial Ti/IrRu as the working electrode, a platinum coil as the counter electrode, and the Hg/HgO reference electrode. The electrolyte was 0.5 M NaCl. The as-prepared α-$Co(OH)_2$ powder (30 mg) was dispersed in the electrolyte solution (12.5 mL) and added to the anolyte compartment. An anodic current was applied to generate the oxidizing species (Cl_2) in situ via the chlorine evolution reaction (CER). Electrolysis continued until the total charge exceeded 36 C to ensure complete conversion of the precursor to γ-CoOOH. The resulting γ-CoOOH was collected and washed with deionized water and anhydrous ethanol and then dried under vacuum at 25 °C.

Electrochemical synthesis of β-CoOOH (Echem-β-CoOOH). Echem-β-CoOOH was synthesized via electrochemical oxidation using oxygen evolution reaction (OER). The electrochemical cell consisted of the commercial Ti/IrRu electrode as the working electrode, a platinum coil as the counter electrode, and the Hg/HgO reference electrode. The electrolyte was 0.5 M NaCl, which had been adjusted to pH 14. The as-prepared α-$Co(OH)_2$ powder (30 mg) was dispersed in the electrolyte solution (12.5 mL) and added to the anolyte compartment. An anodic current was applied to generate in-situ oxidizing species (O_2) via the OER. Electrolysis continued until the complete conversion of the precursor to β-CoOOH. The suspension was centrifuged, and thoroughly washed to eliminate any residual materials. Subsequently, the product was subjected to vacuum drying at 25 °C.

Chemical synthesis of γ-CoOOH (Chem-γ-CoOOH). The pre-synthesized α-$Co(OH)_2$ powder (30 mg) was dispersed in a mixture of 0.5 M NaOH (1.7 mL) and NaOCl (5.2wt%, 20 mL) and heated at 50 ˚C for an hour under atmospheric conditions. The suspension was centrifuged, and thoroughly washed with deionized water and anhydrous ethanol. Subsequently, the product was subjected to vacuum drying at 25 °C.

Chemical synthesis of β-CoOOH (Chem-β-CoOOH). The pre-synthesized α-$Co(OH)_2$ powder (30 mg) was dispersed in a mixture of 0.5 M NaOH (1.7 mL) and NaOCl (5.2wt%, 0.5 mL) and heated at 50 °C for an hour under atmospheric conditions. The suspension was centrifuged, and thoroughly washed with deionized water and anhydrous ethanol. Subsequently, the product was subjected to vacuum drying at 25 °C.

Chemical synthesis of $Mn(OH)_2$. $Mn(OH)_2$ was synthesized via a controlled precipitation method under inert conditions. The precursor solution was prepared by dissolving manganese (II) chloride tetrahydrate ($MnCl_2·4H_2O$, 0.2 g) in deionized water (2 mL). The solution was then purged with argon gas (30 min) to remove dissolved oxygen. Following the purging process, NaOH solution (0.5 M, 1 mL) was rapidly injected into the solution under continuous stirring for 5 minutes at room temperature. The resulting precipitate was collected via centrifugation and washed once with the NaOH solution (0.5 M). The final product was isolated and stored under inert conditions to prevent oxidation.

Electrochemical synthesis of δ-MnO_2. The electrochemical synthesis was conducted in a three-electrode cell. The Ti/IrRu served as the working electrode, a platinum coil served as the counter electrode, and the Hg/HgO electrode was employed as the reference electrode. The electrochemical reactions were carried out in the NaCl solution (0.5 M), with the pH adjusted to 14 using NaOH to promote the desired oxidation pathway. The as-prepared $Mn(OH)_2$ was dispersed in the NaCl solution and added to the anolyte compartment. An anodic current was

applied to the working electrode to initiate the OER. This electrochemical process generated in-situ oxygen gas, which served as the oxidant for the conversion of $Mn(OH)_2$ to δ-MnO_2. The collected material was washed several times with deionized water to eliminate residual electrolyte and any soluble by-products. The purified δ-MnO_2 was then dried under vacuum at an ambient temperature for 2 hours to yield the final product as a powder.

Electrochemical synthesis of γ-MnO_2. The electrochemical synthesis was conducted in a three-electrode cell. The Ti/IrRu served as the working electrode, a platinum coil served as the counter electrode, and the Hg/HgO electrode was employed as the reference electrode. The electrochemical reactions were carried out in the NaCl solution (0.5 M). The as-prepared $Mn(OH)_2$ was dispersed in the NaCl solution and added to the anolyte compartment. An anodic current was applied to the working electrode to initiate the CER. This electrochemical process generated in-situ chlorine gas, which served as the oxidant for the conversion of $Mn(OH)_2$ to γ-MnO_2. The collected material was washed several times with deionized water to eliminate residual electrolyte and any soluble by-products. The purified γ-MnO_2 was then dried under vacuum at an ambient temperature for 2 hours to yield the final product as a powder.

Electrochemical measurements. The catalysts were loaded on carbon paper (CP) (10 mm × 10 mm), which served as the working electrode. Electrocatalyst powder (4 mg) was dispersed in a mixture of DI water/ethanol (1:3(v/v), 1 mL) with Aquivion solution (20 μL, 25wt%, Sigma-Aldrich). The prepared ink was drop-casted onto the carbon paper surface (0.22 mg cm^{-2}), and then dried for at least 30 min. All electrochemical properties were measured using a potentiostat (CHI604 Austin, Ivium V89128 Vertex One). Hg/HgO and Pt coil served as the reference and counter electrodes, respectively. The electrochemical measurements were performed in 1 M KOH solution for the water oxidation. The obtained electrodes were initially cycled five times using cyclic voltammetry (CV) until a stable peak was obtained. The

electrochemical properties were assessed using CV, linear sweep voltammetry (LSV), chronoamperometry, Tafel slope analysis, electrochemical impedance spectroscopy, chronoamperometry, and chronopotentiometry. Chronopotentiometry was conducted under the current densities of 10, 20, 50, 100 mA cm^{-2}, followed by reverse currents. Electrochemical surface area (ECSA) values for samples were calculated by double-layer capacitance measured with the CV method in a non-faradaic potential window with scan rates (v) of 10, 20, 30, 40, and 50 mV s^{-1}. From the difference of average currents for forward and reverse sweeps, capacitive currents (I_{dl}) were taken. Then, double-layer capacitance (C_{dl}) was given by the slope of a linear fit for I_{dl} versus V graph.

The hydrogen gas was analyzed using a gas chromatograph (6500GC System; YL Instrument Co.) equipped with a Carboxen-1000 column, thermal conductivity detector, flame ionization detector, and methanizer, with Ar (99.999%) as the carrier gas. Due to the two-electron process of the production of H_2, the Faradaic efficiency of H_2 was estimated using the following equation:

$$\textbf{Faradaic efficiency} = 2 \times F \times n_i / (I \times t)$$

where F = Faraday's constant (96485 C mol^{-1}), n_i = moles of H_2, I = current (A), t = time (s).

Quantitative titration of Cl_2/HOCl. The electrochemically generated HOCl was quantified via indirect analysis using a triiodide formation method. HOCl was electrochemically produced using a commercial Ti/IrRu dimensionally stable anode (DSA) as the working electrode. The electrolyte was a NaCl solution (0.5 M, 12.5 mL, pH 7). Electrochemical measurements were performed in an H-type cell under ambient conditions, utilizing a CHI 604E potentiostat with the Hg/HgO reference electrode. To ensure homogeneous reaction conditions, the electrolyte was continuously agitated at 400 rpm using a magnetic stirrer bar. This analytical approach

employed two reagent solutions, R1, composed of potassium iodide (KI), and R2, composed of glacial acetic acid as an iodine scavenger. The concentration of HOCl was determined by its stoichiometric reaction with these reagents, which enabled the precise quantification of the electrocatalytically generated species. The production of HOCl in the electrochemical progress was detected as the following chemical reaction:

$$KI \rightarrow K^+ + I^-$$

$$HOCl + H^+ + 2I^- \rightarrow I_2 + Cl^- + H_2O$$

$$I_2 + I^- \rightleftharpoons I_3^-$$

The strong absorbance of triiodide at $\lambda_{352\ nm}$ was used to quantify the formation of HOCl.

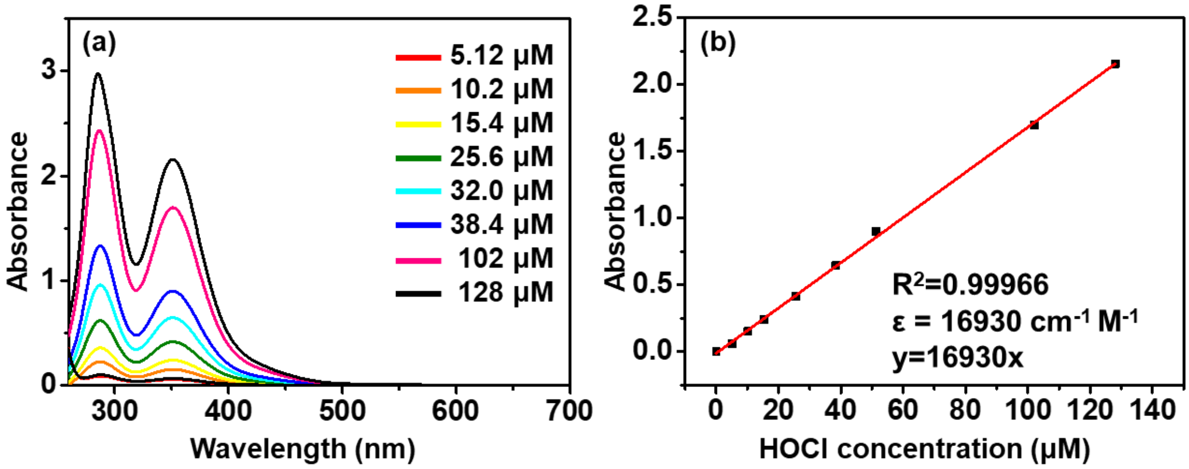

Quantitative titration of Cl_2/HOCl. The faradaic efficiency of the production of HOCl using the Ti/IrRu anode in NaCl solution (0.5 M, < pH 7) was determined to be 95%. The oxidation of α-$Co(OH)_2$ (30 mg) to γ-CoOOH required a 2:1 molar ratio of α-$Co(OH)_2$ to HOCl. The amount of HOCl that was needed was calculated as follows:

1. Moles of α-$Co(OH)_2$:

$$30\ mg\ /\ (92.95\ g\ mol^{-1}) = 0.3228\ mmol$$

2. Moles of HOCl required:

$$0.3228 \text{ mmol} / 2 = 0.1614 \text{ mmol}$$

3. The charge required to produce the equivalent amount of HOCl was calculated using the following equation:

$$Q = Zn_iF = 2 \times (1.6\times10^{-4} \text{ mol}) \times (96{,}485 \text{ C mol}^{-1}) / 0.95 = 32.78 \text{ C}$$

Q = charge (C), n_i = moles of HOCl, F = Faraday constant (96,485 C mol^{-1})

1. Supplementary Tables

Supplementary Table 1. Structural parameters of the cobalt hydroxides, α-$Co(OH)_2$, Echem-β-CoOOH, and Echem-γ-CoOOH.

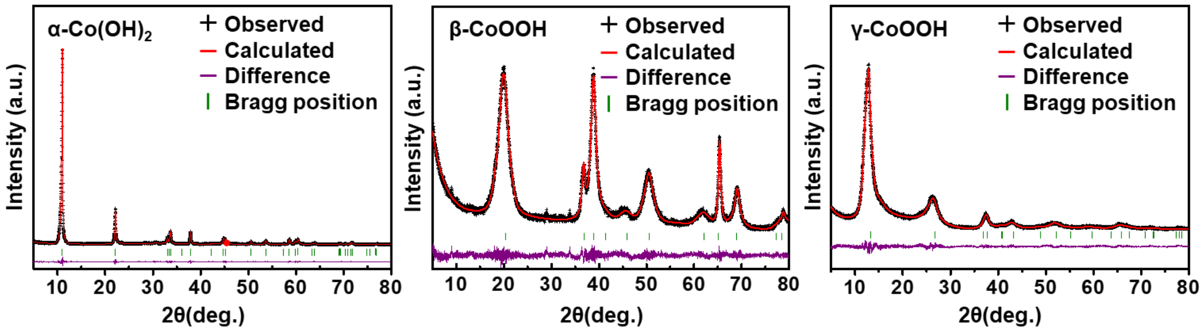

Trigonal<br>α (= β) = 90.0°, γ = 120.0°		R_{wp} [%]	R_p [%]	a (= b) [nm]	c [nm]	GOF
$R\bar{3}m$ (No. 166)	α-$Co(OH)_2$	11.6	7.55	0.3149(4)	2.408(3)	1.09
$P\bar{3}m1$ (No. 164)	β-CoOOH	6.99	5.05	0.2863(4)	1.309(2)	1.21
$R3m$ (No. 160)	γ-CoOOH	7.35	5.20	0.2834(2)	1.995(2)	1.24

Supplementary Table 2. Structural parameters of γ-CoOOH refined using the XRD data acquired at 297 K. The symbols, B_{eq} and g, represent the isotropic thermal parameter and the occupation factor, respectively. The numbers in parentheses are the estimated standard deviations of the last significant figure.

- R- factors: R_{wp} = 7.35%, R_p = 5.20%, R_e = 2.14%, GOF (goodness of fit) = 1.24

- Lattice parameter: a (= b) = 0.2834(2) nm, c = 1.995(2) nm, α (= β) = 90°, γ = 120°

- Space group: R 3 m (No. 160), Z = 3

**Atom**	**Site**	***x***	***y***	***z***	***g***	B_{eq}/Å^2
Co_{slab}	3a	0.0	0.0	0.0	1.0	1.98(1)
O_{1_slab}	3a	0.0	0.0	0.3756(1)	1.0	2.33(2)
O_{2_slab}	3a	0.0	0.0	0.6154(1)	1.0	2.54(2)
$Na_{interslab}$	3a	0.0	0.0	0.1802(1)	0.381(3)	3.76(2)
$O_{1\_interslab}$	3a	0.0	0.0	0.1404(1)	0.283(3)	3.48(2)

2. Supplementary Figures

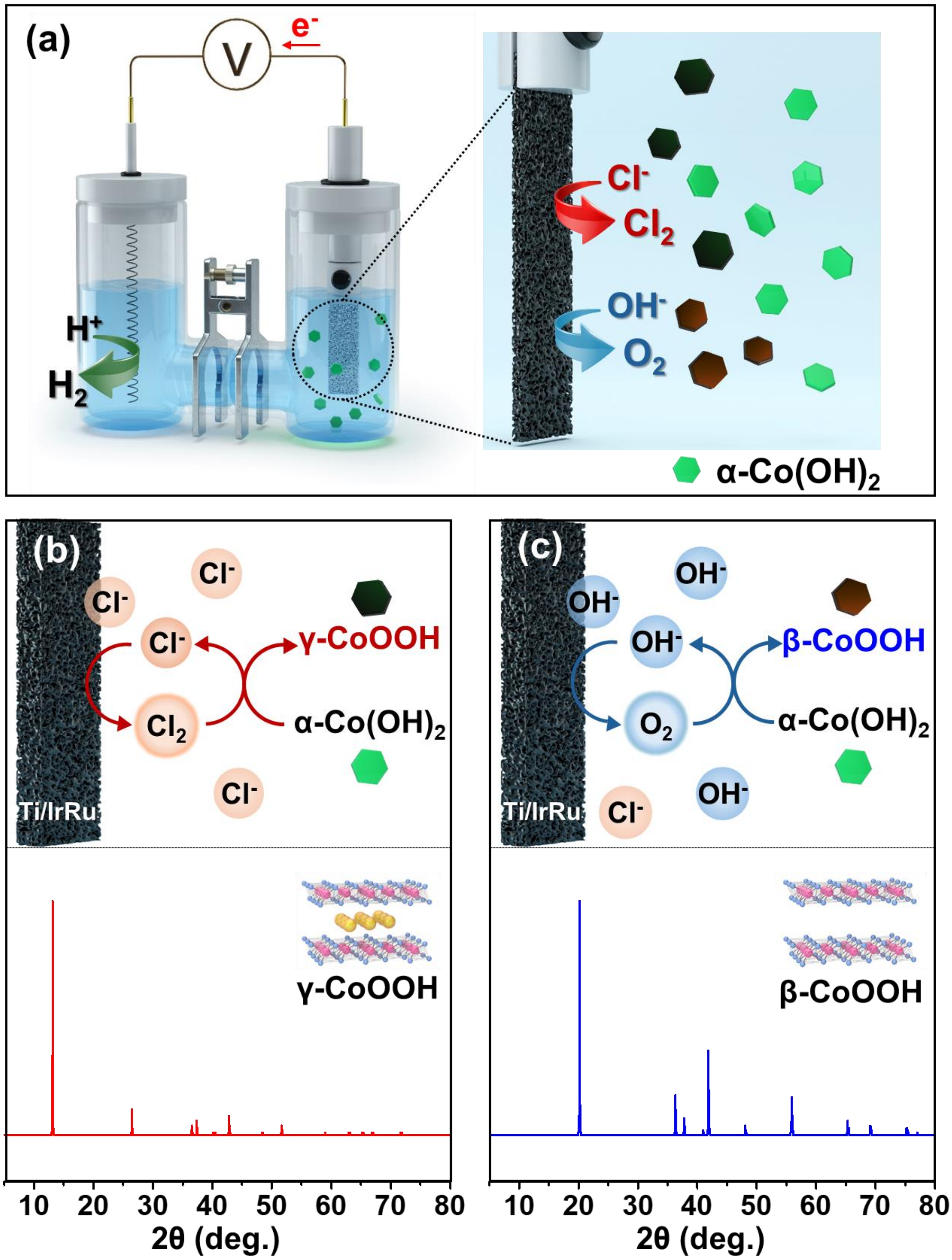

Figure S1. (a) Schematic of the oxidative transition from α-$Co(OH)_2$ to CoOOH via in-situ generation of tailored oxidants, with details of the oxidative conversion to (b) γ-CoOOH and (c) β-CoOOH crystals by Cl_2 and O_2, respectively. Simulated XRD patterns in (b) and (c) were obtained by rietveld refinement.

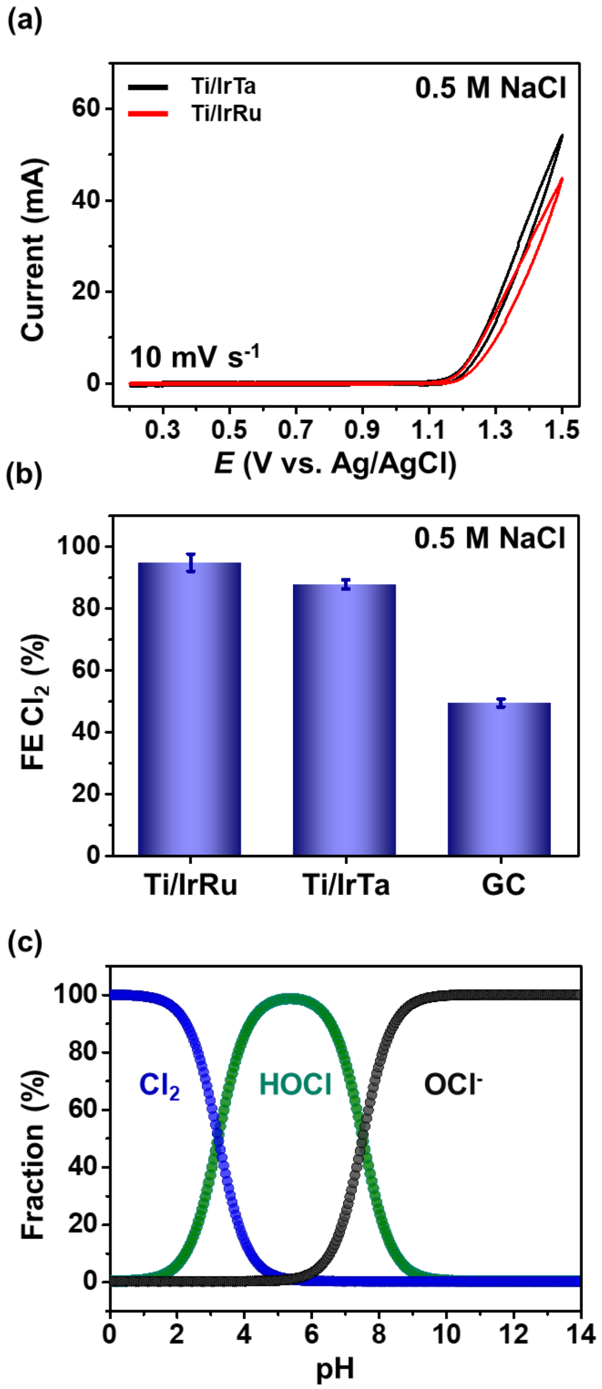

Figure S2. (a) CVs of each of the metal-based electrodes in 0.5 M NaCl (scan rate: 10 mV s^{-1}), (b) FE of Cl_2 for the DSA and glassy carbon (GC) electrodes, respectively. The DSA electrodes are composed of Ti/IrRu, and Ti/IrTa, respectively. For the FE measurements, a potential of 1.25 V vs Ag/AgCl was applied to the DSA electrodes, while 1.8 V was applied to the GC electrode. (c) Fraction of aqueous active chlorine species as a function of pH, calculated using a composition of 0.5 M NaCl.

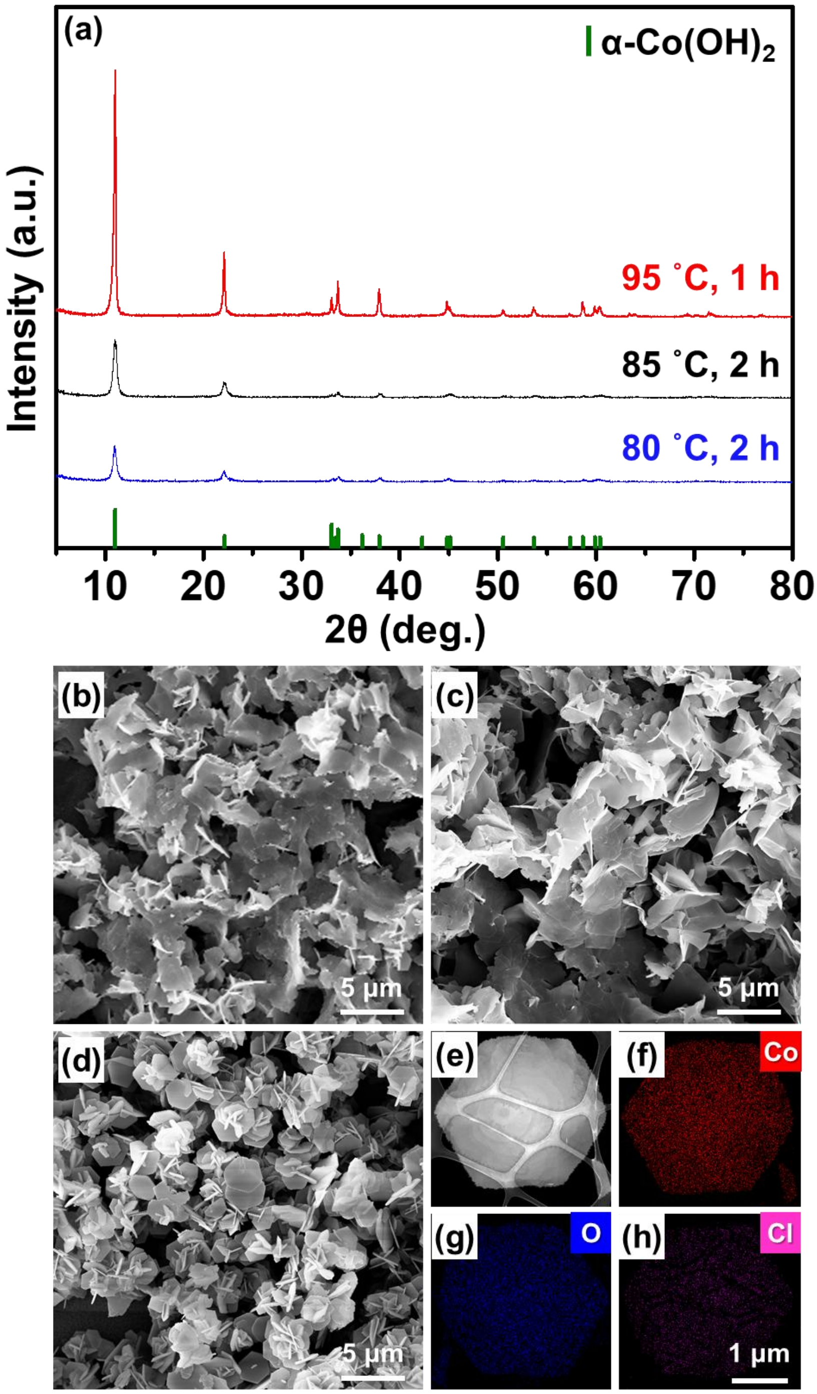

Figure S3. Characterization of α-$Co(OH)_2$. (a) XRD patterns of the sample prepared at various reaction temperatures and times. SEM images of the sample, synthesized at (b) 80 °C for 2 h, (c) 85 °C for 2 h, and (d) 95 °C for 1 h. (e) HAADF-STEM images and TEM-EDX elemental mapping of (f) Co, (g) O, and (h) Cl, respectively.

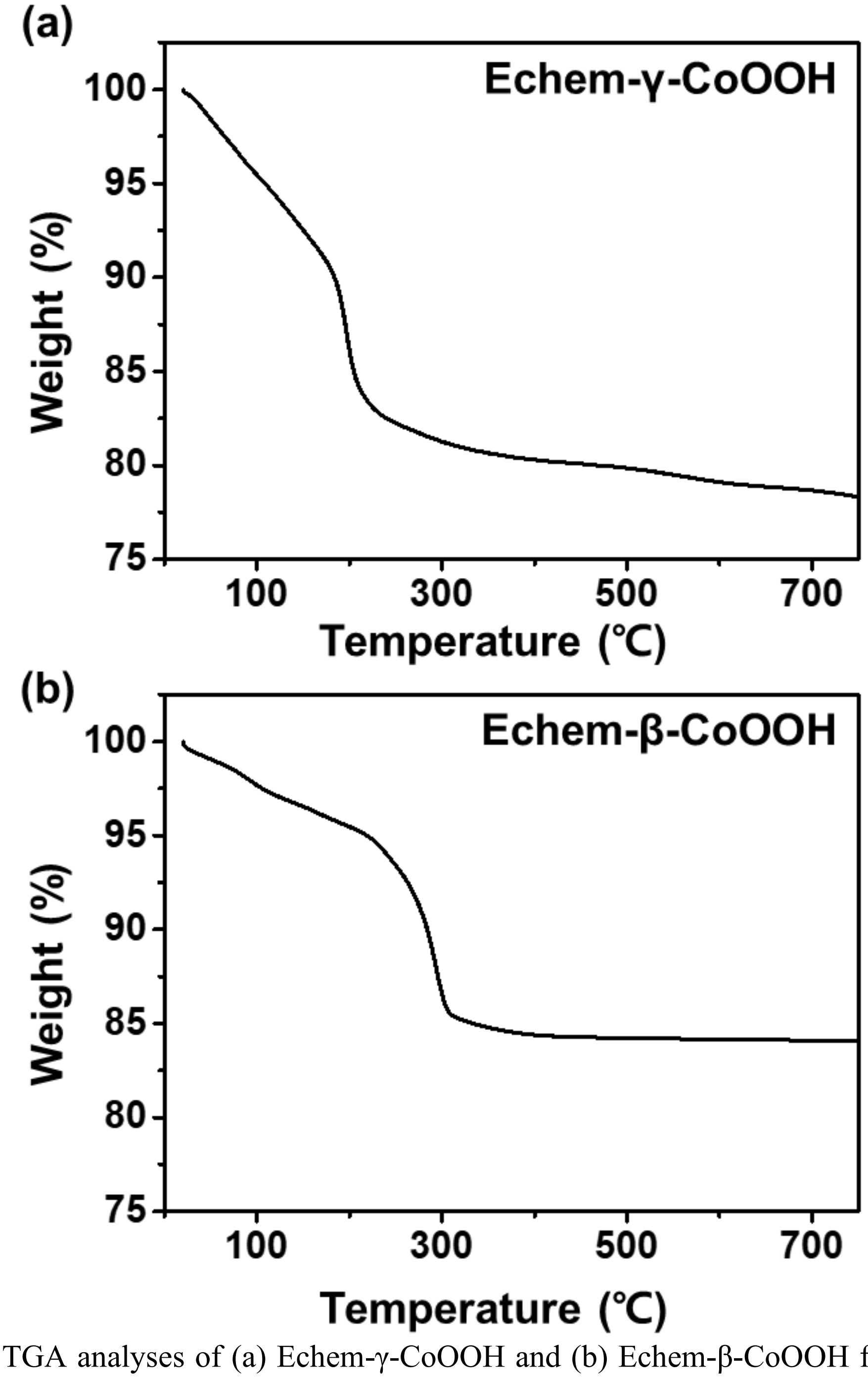

Figure S4. TGA analyses of (a) Echem-γ-CoOOH and (b) Echem-β-CoOOH from 25 °C to 750 °C in air.

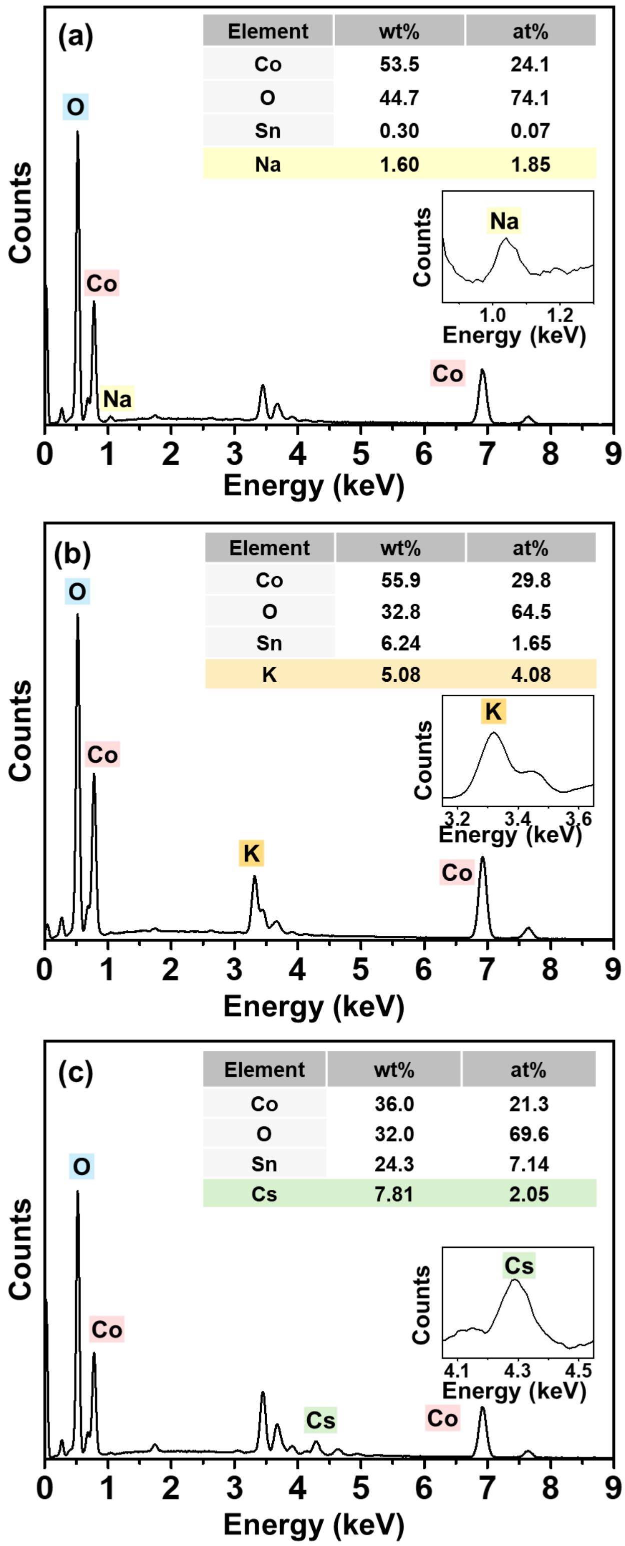

Figure S5. SEM-EDX spectra of Echem-γ-CoOOH. The table in the inset presents the concentration of each element (wt% and at%). The graph in the inset shows the cation peak on the SEM-EDX spectrum, which means cations are intercalated into Echem-γ-CoOOH.

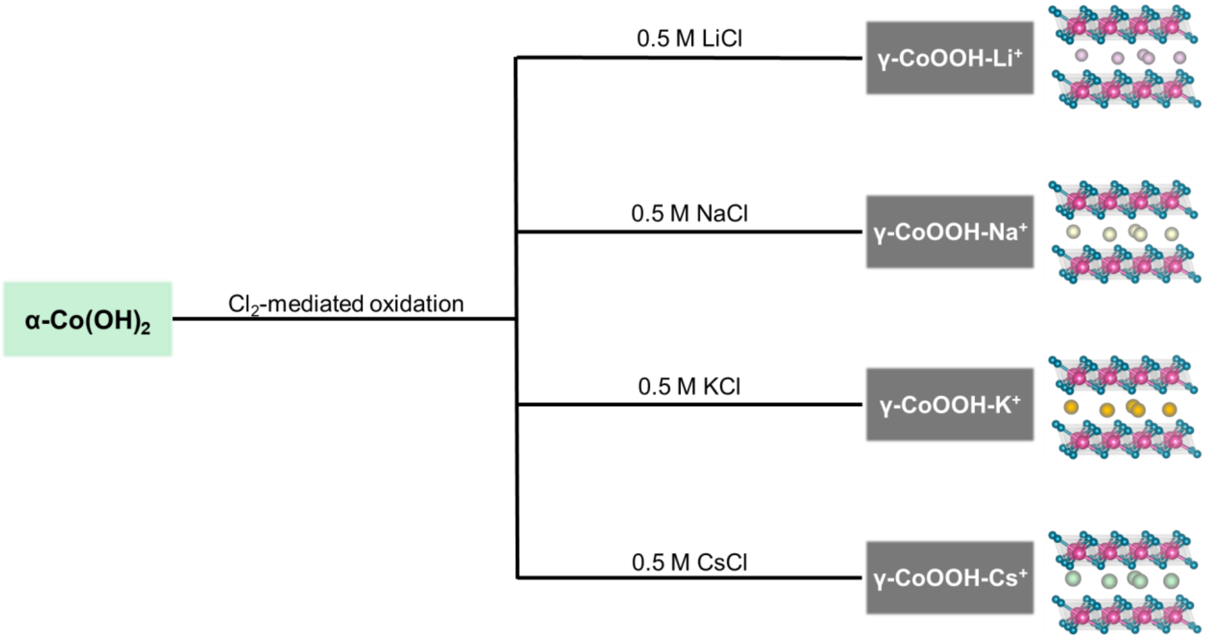

Figure S6. Schematic illustration of metal cation intercalation and the resulting elongation of the Co-O bonds in Echem-γ-CoOOH.

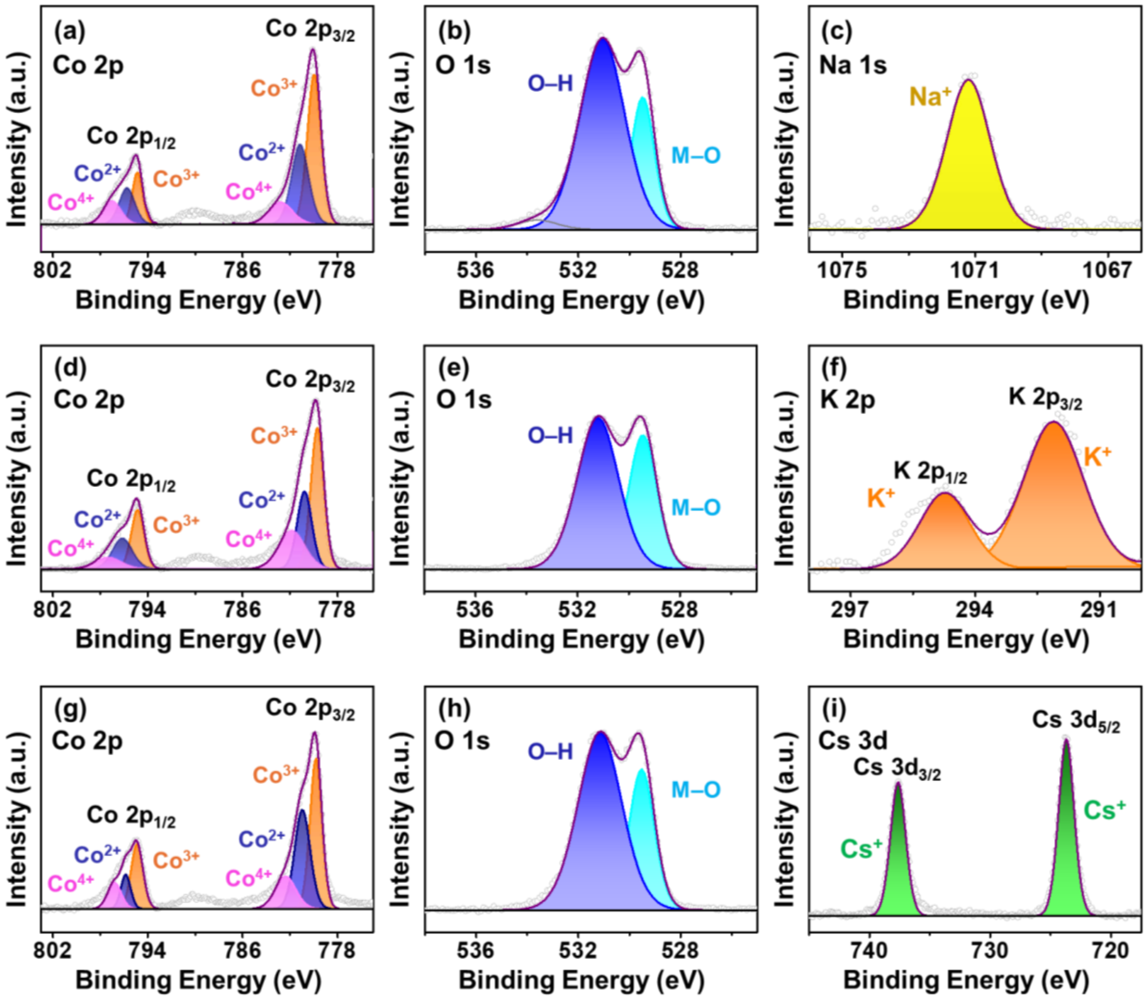

Figure S7. XPS analyses of γ-CoOOH samples intercalated with (a–c) Na^+, (d–f) K^+, and (g–i) Cs^+ ions.

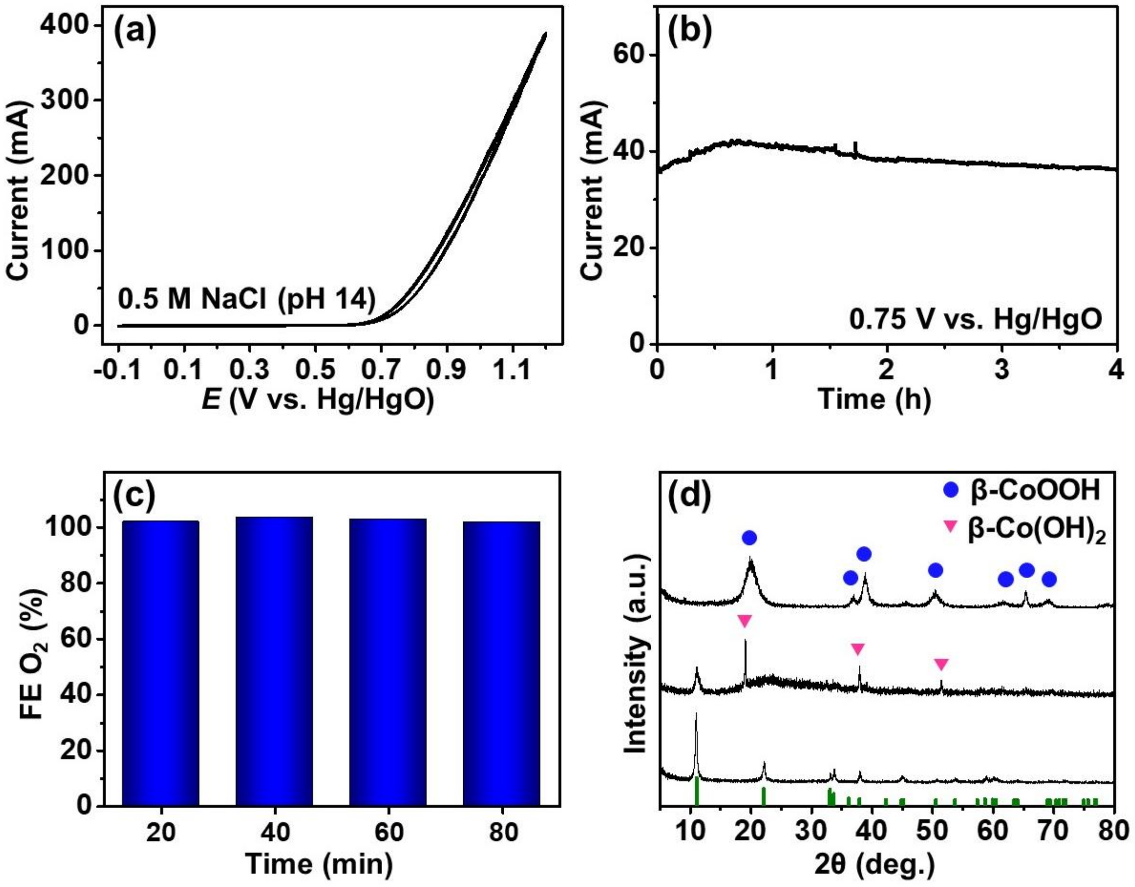

Figure S8. (a) CV (scan rate: 10 mV s^{-1}) using a commercial Ti/IrRu electrode in 0.5 M NaCl and 2 M NaOH in the presence of α-$Co(OH)_2$ microplates. (b) Chronoamperometry measurement and (c) FE of O_2 to the applied charge at 0.75 V vs. Hg/HgO in 0.5 M NaCl and 2 M NaOH with α-$Co(OH)_2$ microplates. (d) XRD analyses as a function of the generated O_2 concentration.

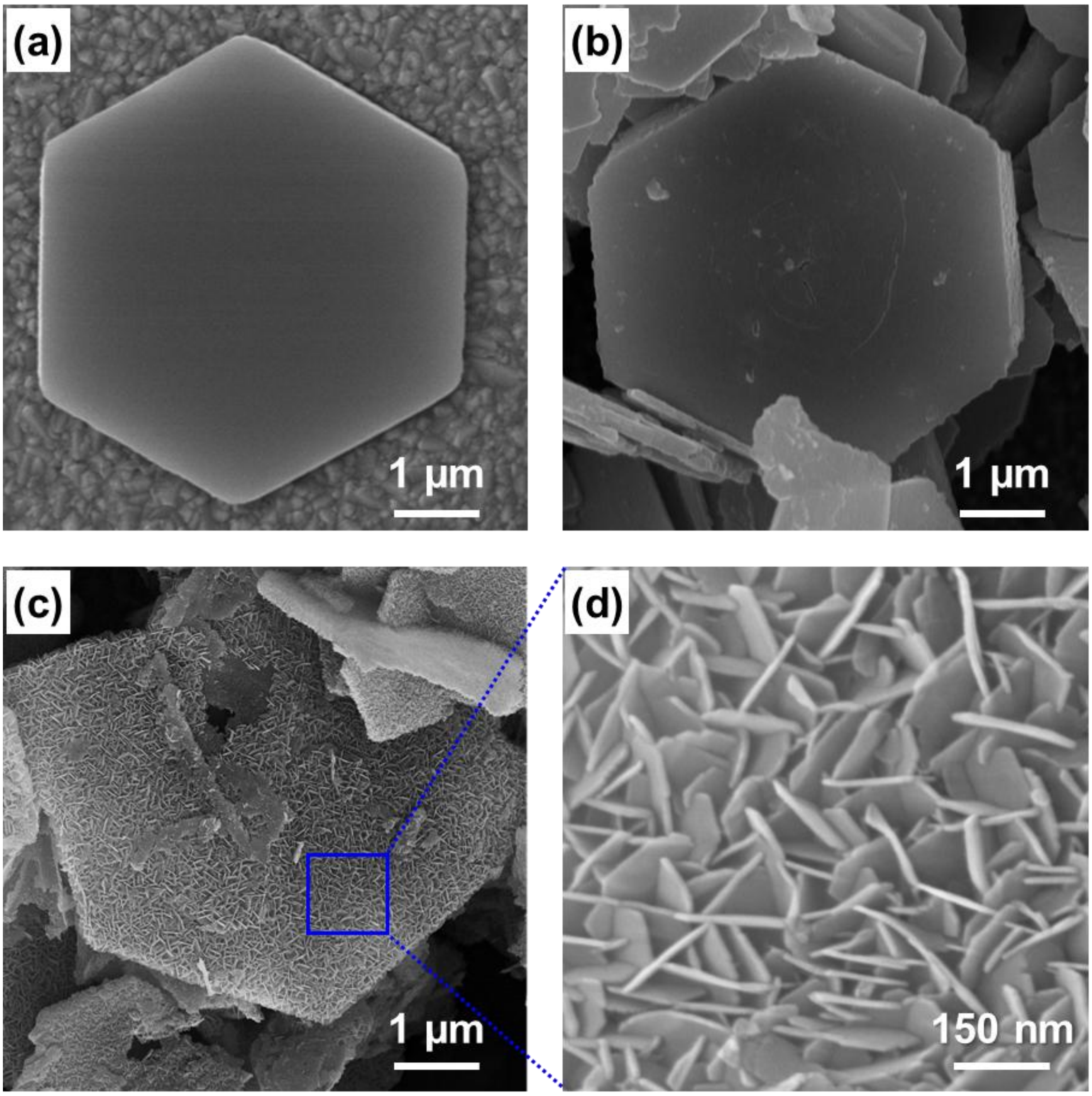

Figure S9. SEM images of (a) α-$Co(OH)_2$, (b) Echem-γ-CoOOH, and (c), (d) Echem-β-CoOOH.

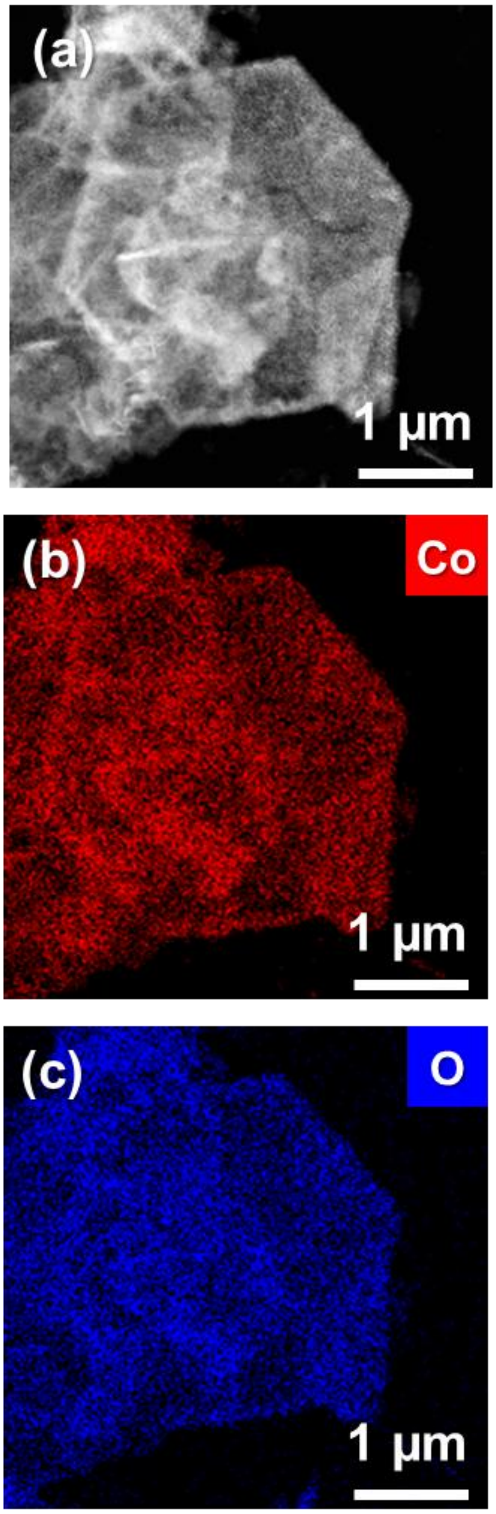

Figure S10. HAADF-TEM images of Echem-β-CoOOH. (b) Elemental map corresponding distributions of (b) Co, and (c) O, respectively.

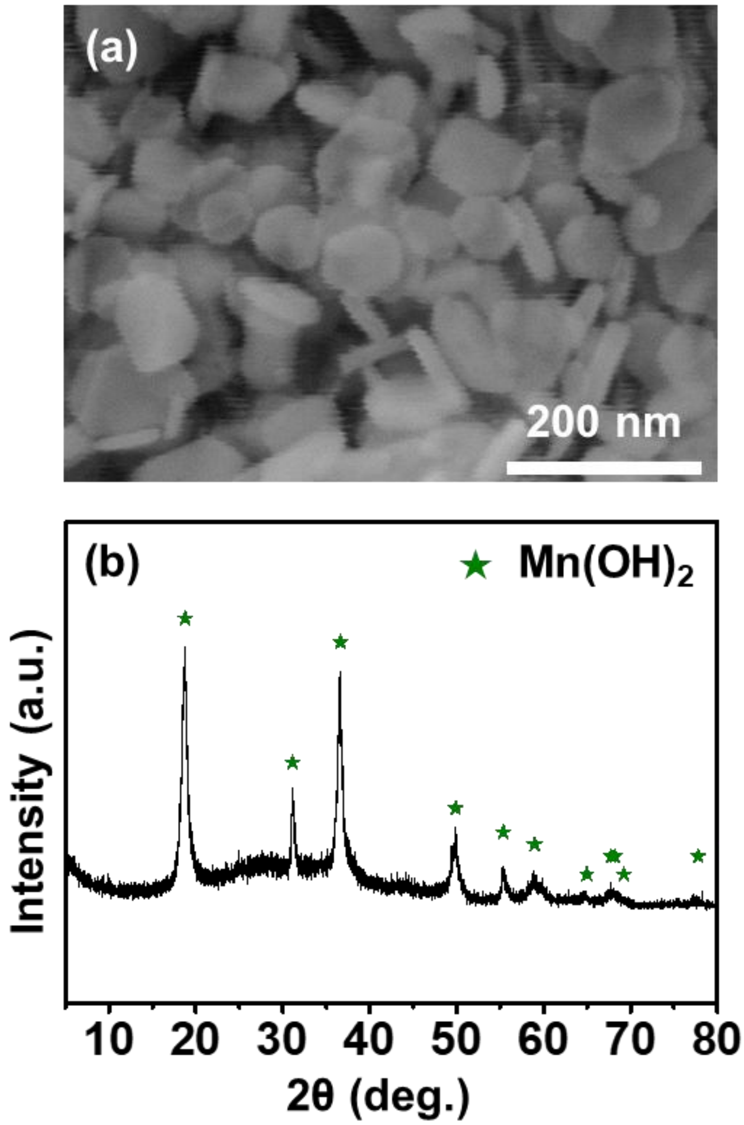

Figure S11. (a) SEM image of $Mn(OH)_2$ nanoparticles, revealing their plate-like morphology and (b) XRD analysis of $Mn(OH)_2$, revealing the brucite structure.

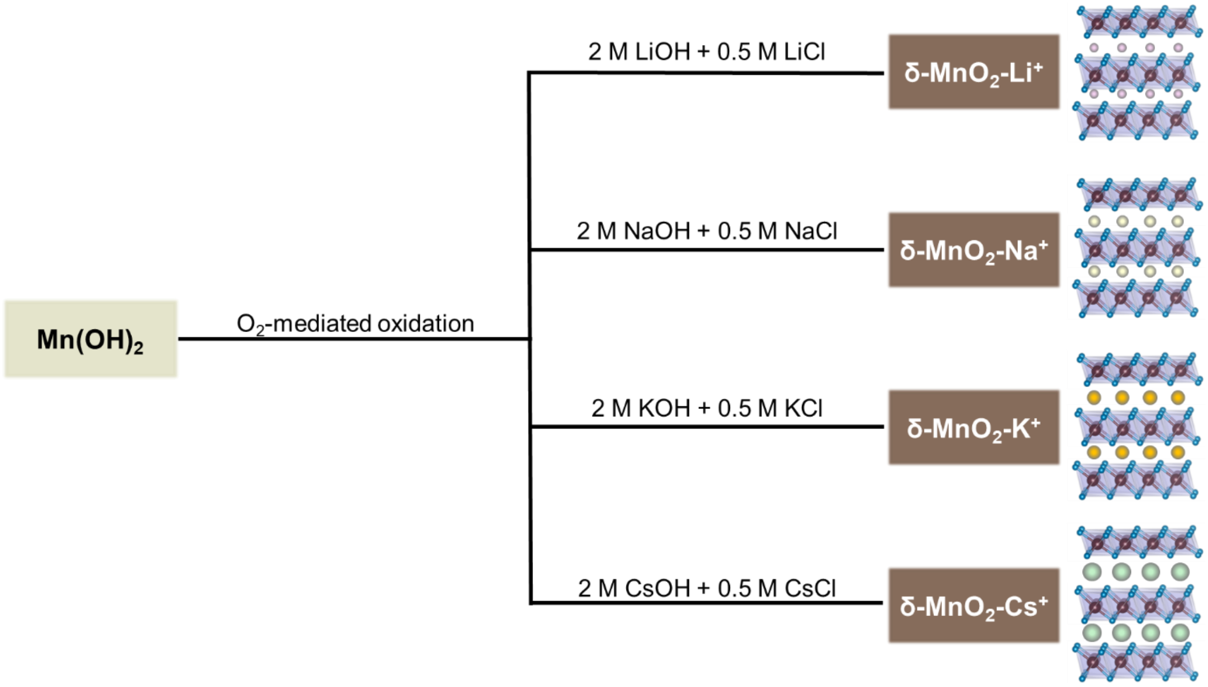

Figure S12. Schematic illustration of metal cation intercalation in the layered δ-MnO_2 structure.

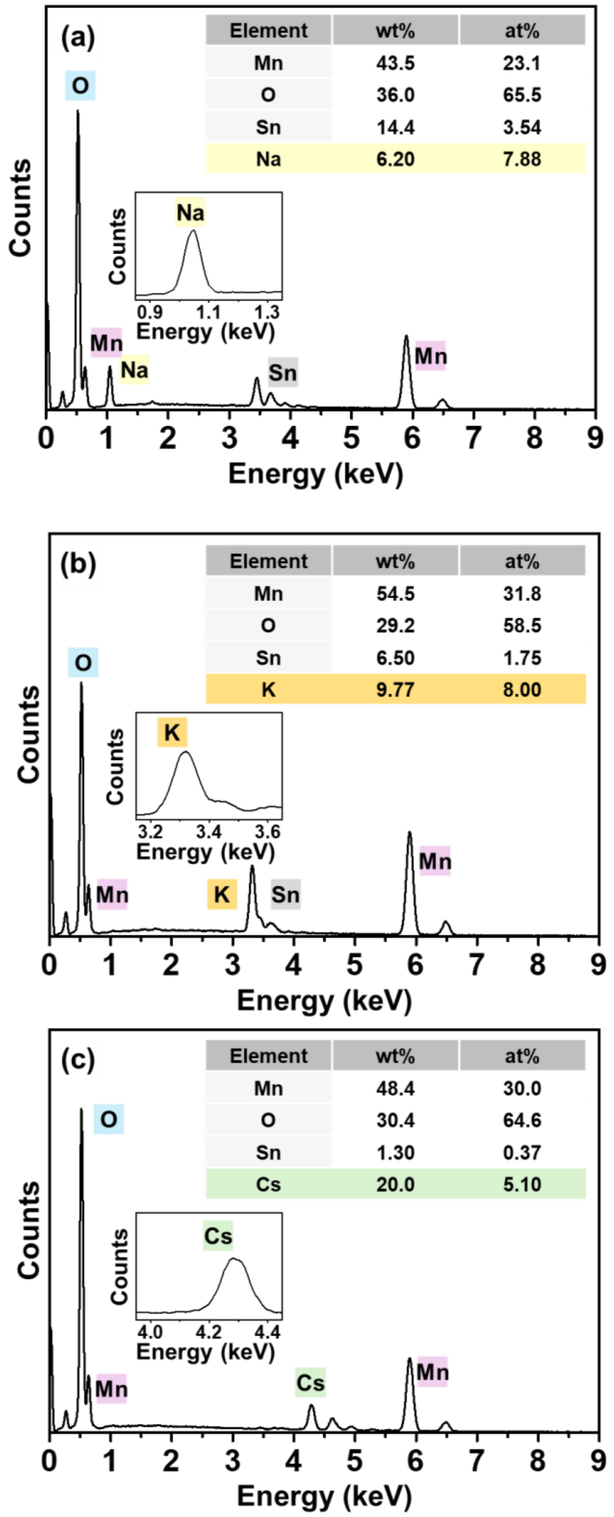

Figure S13. SEM-EDX spectrum of δ-MnO_2. Inset: tabulated concentration of each of the elements (wt% and at%), and graph plotting the intensity of the cation peak on the SEM-EDX spectrum, indicating that cations are intercalated into δ-MnO_2.

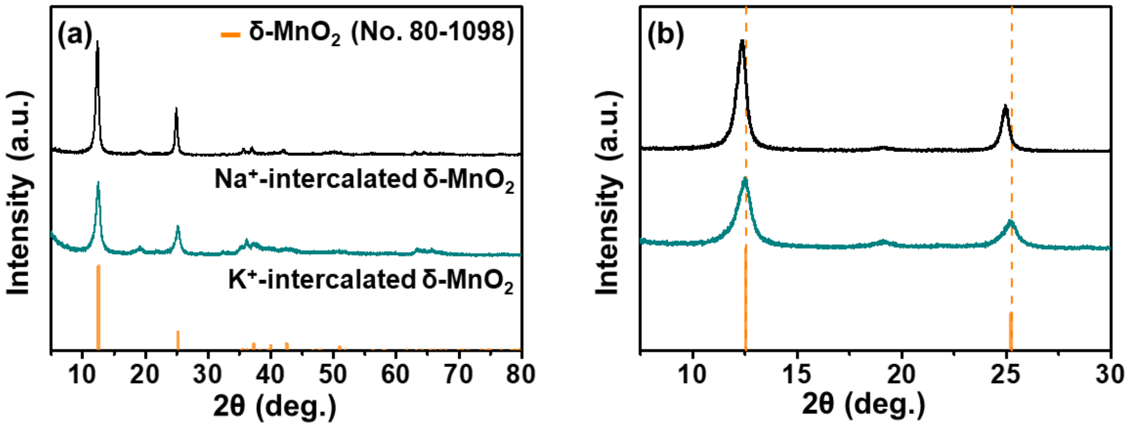

Figure S14. XRD patterns of (a) Na^+ and K^+ intercalated δ-MnO_2 nanosheets, and (b) the extended 2θ region from 10° to 30°.

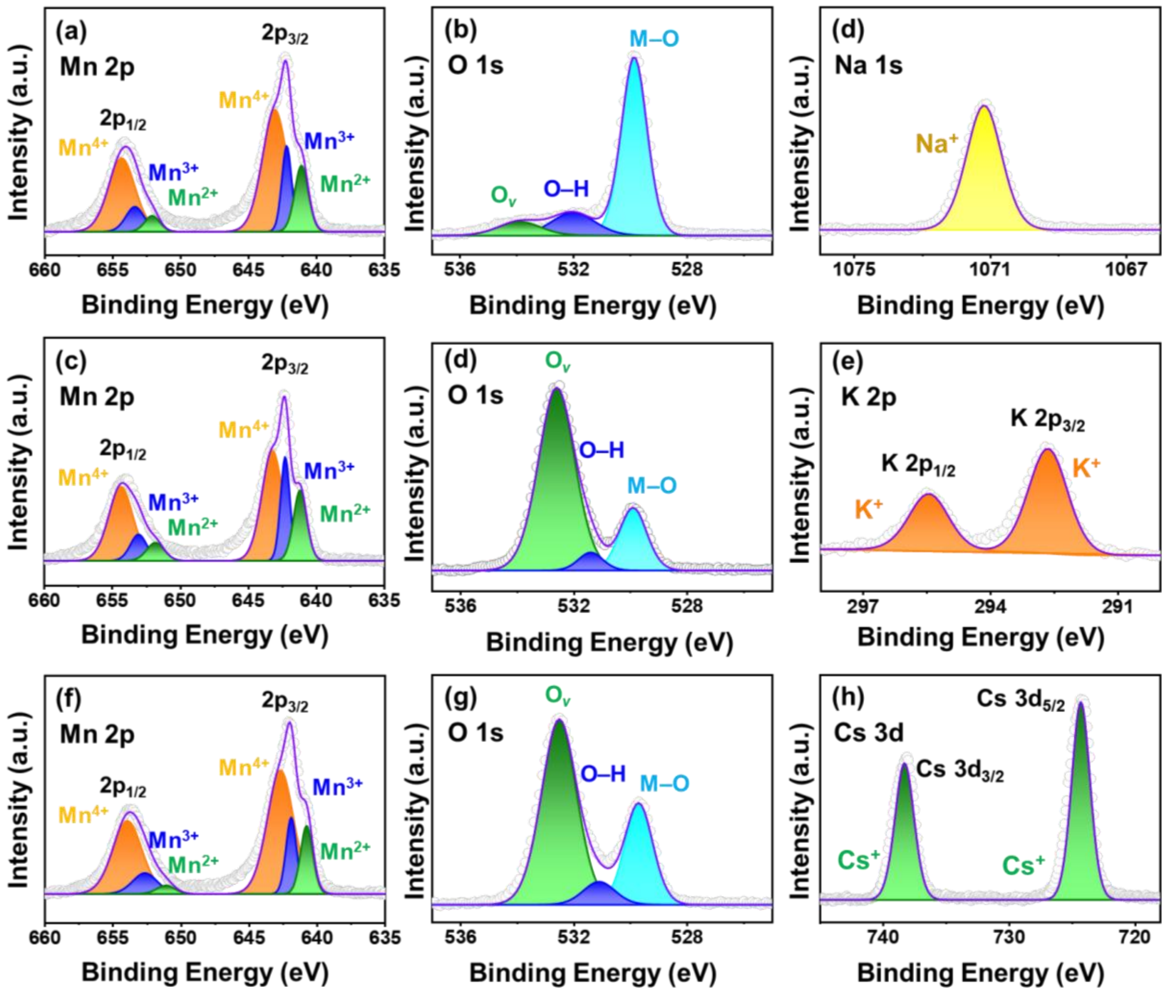

Figure S15. XPS analyses of δ-MnO_2 samples intercalated with (a–c) Na^+, (d–f) K^+, and (g–i) Cs^+ ions.

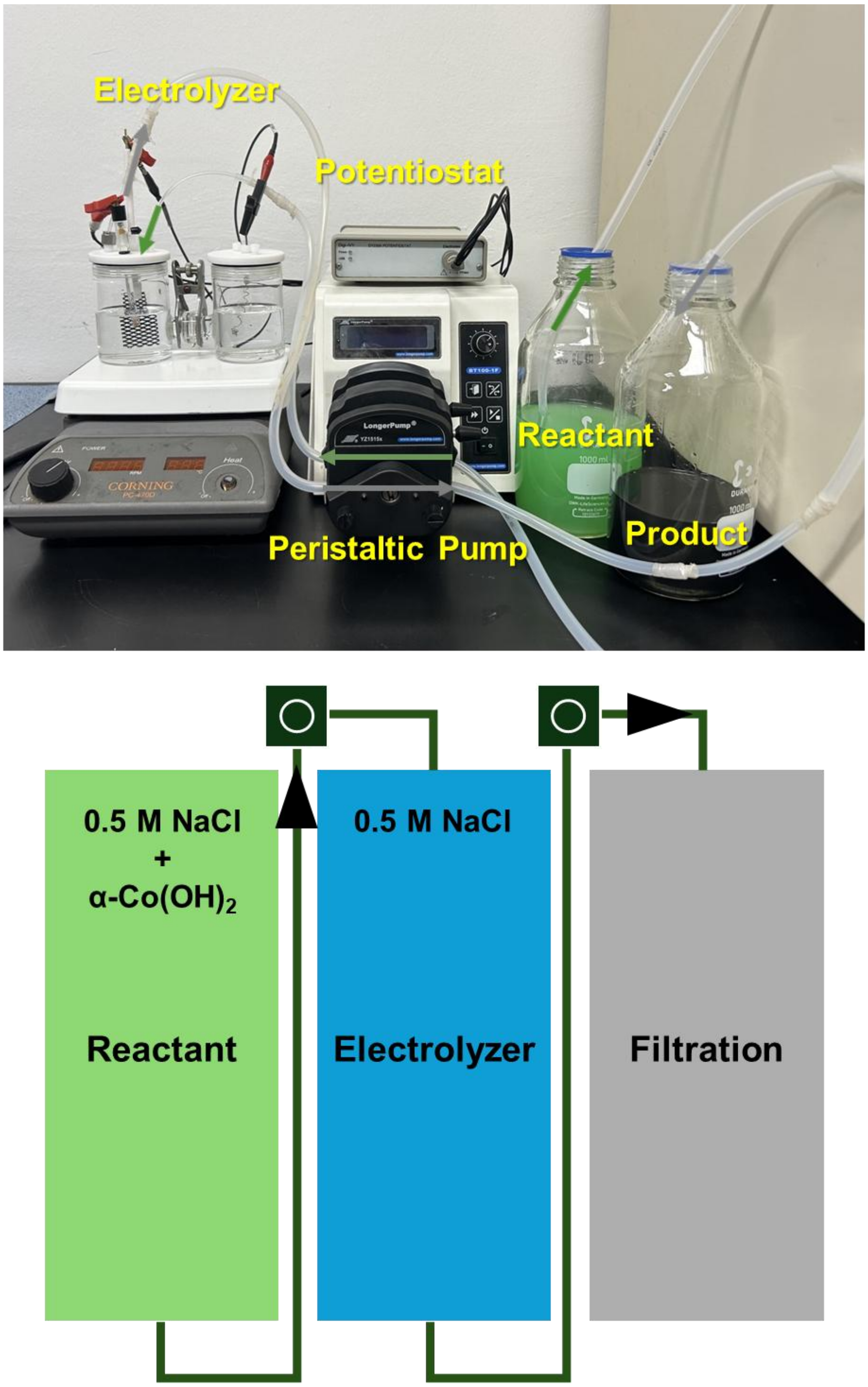

Figure S16. Experimental setup for the electrochemical in-situ generation of oxidizing reagents for the phase-selective synthesis of cobalt oxyhydroxides via the continuous synthesis of a stable and efficient water-splitting catalyst. The flow rate is adjustable from 0.5 to 2.5 sccm, and the applied potential is optimized according to the flow rate.

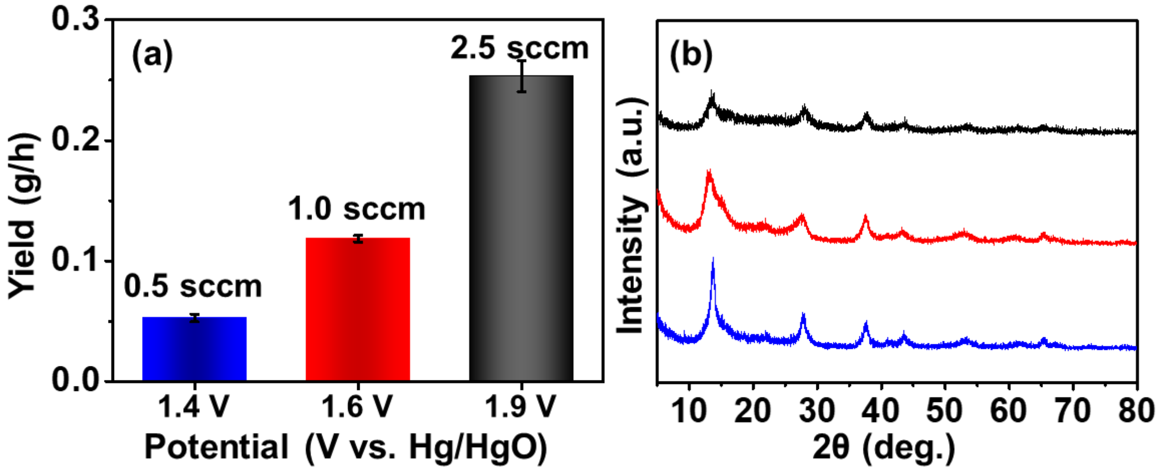

Figure S17. (a) γ-CoOOH powder yields and (b) XRD patterns of synthesized γ-CoOOH powders at different applied potentials and adjusted electrolyte flow rate.

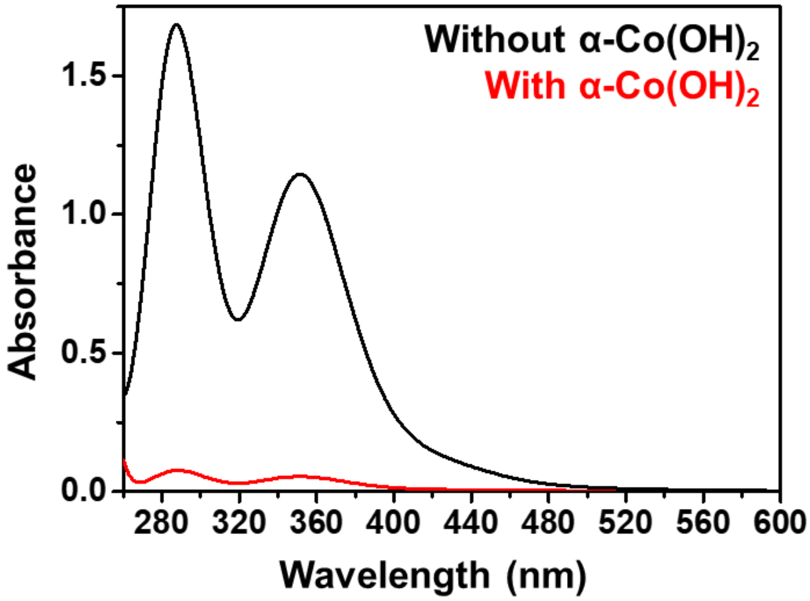

Figure S18. UV–vis absorption spectra of the catholyte after brine electrolysis with (red line) and without (black line) α-$Co(OH)_2$ added to the anode chamber. The spectra were recorded after iodometric treatment, in which residual oxidants were converted to triiodide (I_3^-) for detection.

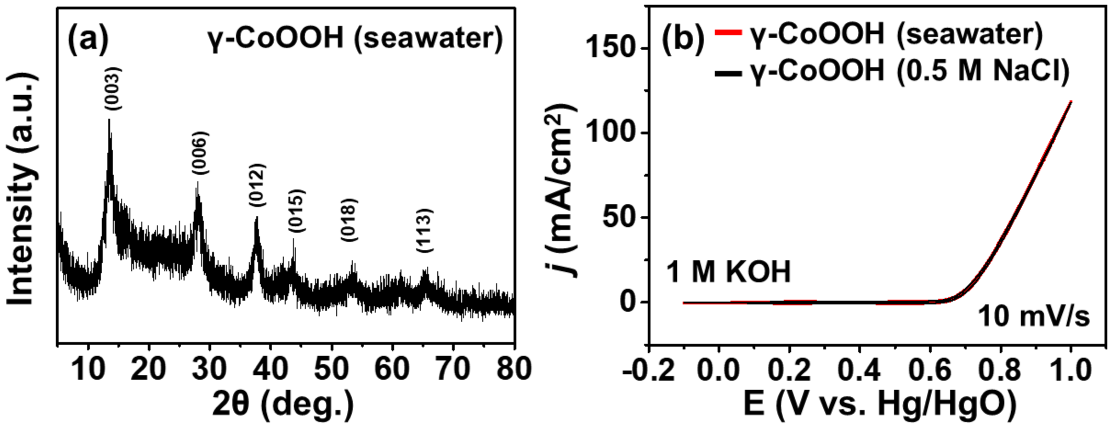

**(c) γ-CoOOH (before)**	Element	Co	O	Na	K	Mg	Ca
	Weight%	59.9	38.80	0.11	0.44	0.32	0.43
	Atomic%	29.2	69.66	0.14	0.33	0.38	0.31

**(d) γ-CoOOH (after)**	Element	Co	O	Na	K	Mg	Ca
	Weight%	51.5	43.1	0.08	5.3	0	0
	Atomic%	23.6	72.7	0.10	3.6	0	0

Figure S19. (a) XRD pattern of γ-CoOOH synthesized in seawater. (b) CVs in 1 M KOH comparing γ-CoOOH synthesized in seawater and in 0.5 M NaCl. SEM-EDX results of γ-CoOOH (c) before and (d) after the OER test in 1 M KOH.

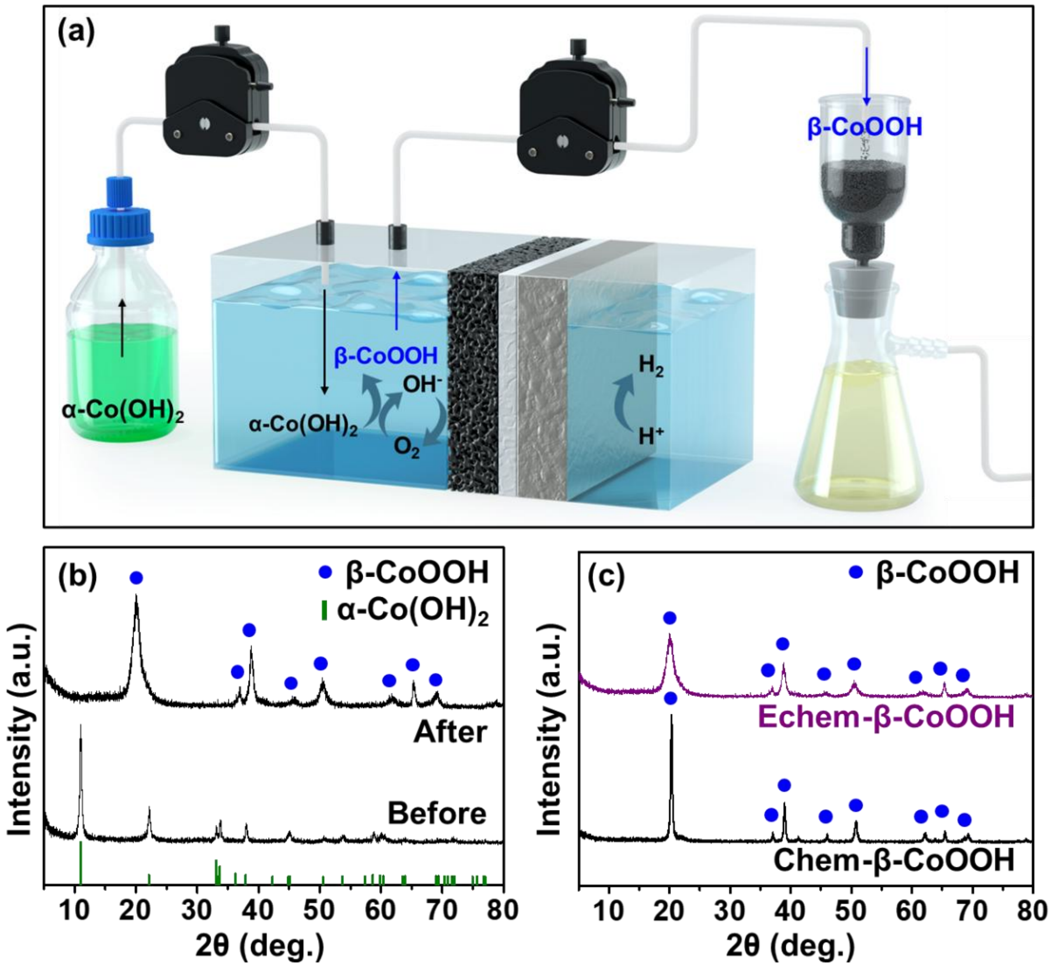

Figure S20. (a) Schematic illustration of electrochemical synthesis of β-CoOOH under flow electrolyte conditions. (b) XRD patterns of the starting material and product. (c) XRD patterns of Echem-β-CoOOH and Chem-β-CoOOH.

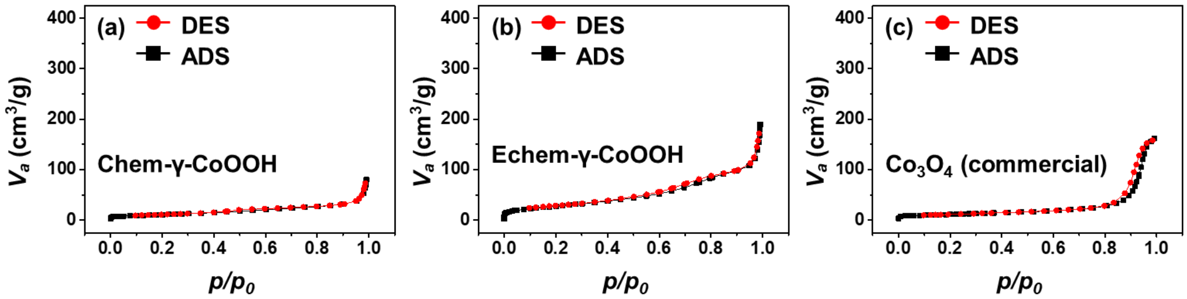

(d) Sample	V_m [$cm^3\ g^{-1}$]	$a_{s,BET}$ [$m^2\ g^{-1}$]
Chem-γ-CoOOH	9.18	40.0
Echem-γ-CoOOH	23.2	101
Co_3O_4 (commercial)	9.17	40.0

Figure S21. Specific surface areas of the samples based on the BET method.

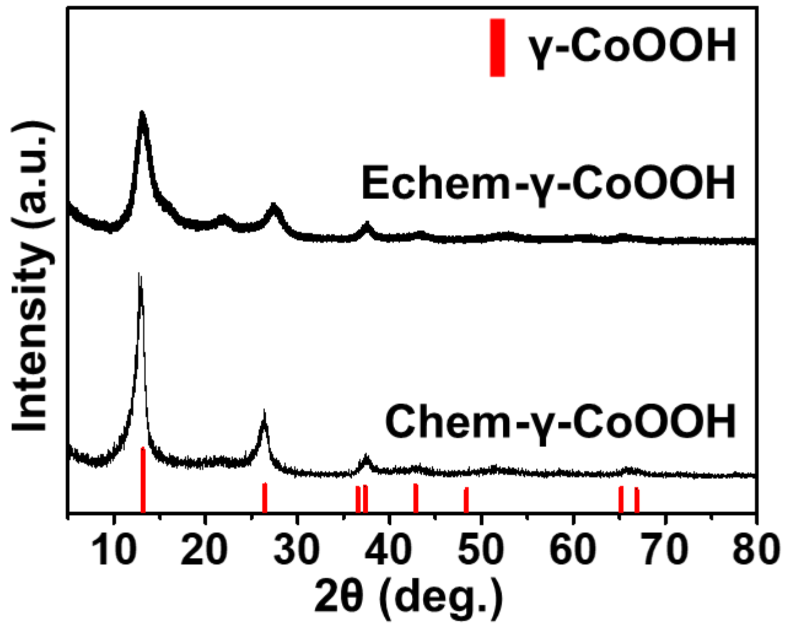

Figure S22. XRD patterns of Echem-γ-CoOOH and Chem-γ-CoOOH samples.

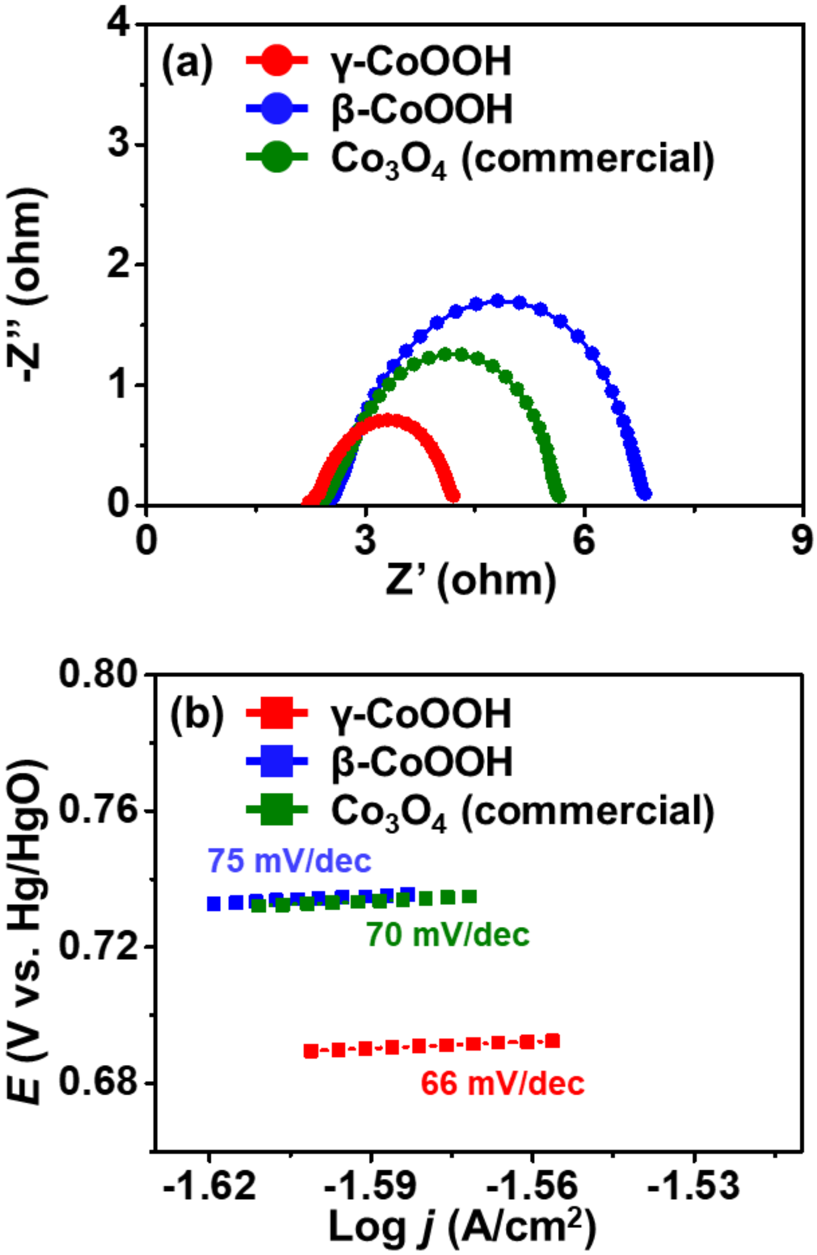

Figure S23. (a) Nyquist plots at 0.70 V vs. Hg/HgO, and (b) Tafel slopes of Echem-γ-CoOOH, Echem-β-CoOOH, and commercial Co_3O_4 in 1 M KOH.

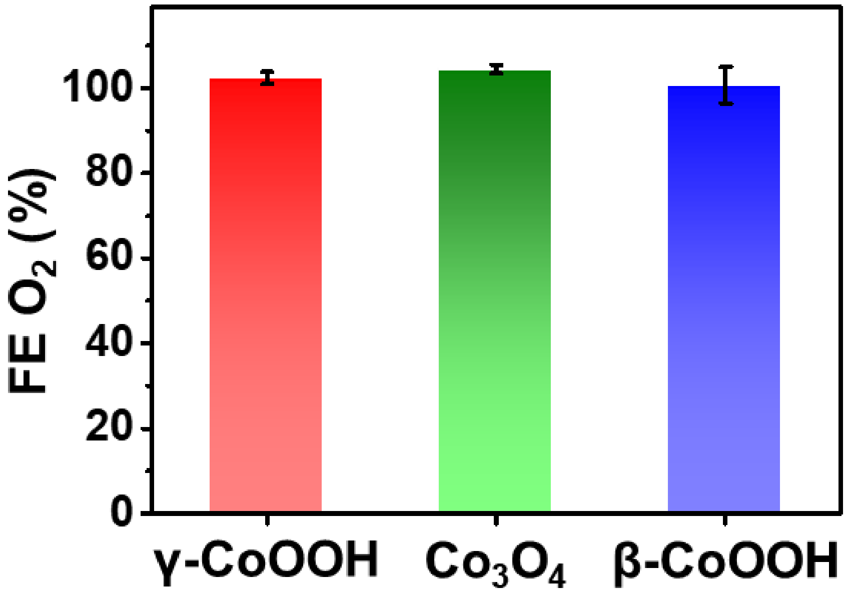

Figure S24. FE of O_2 at 0.75 V vs. Hg/HgO in 1 M KOH.

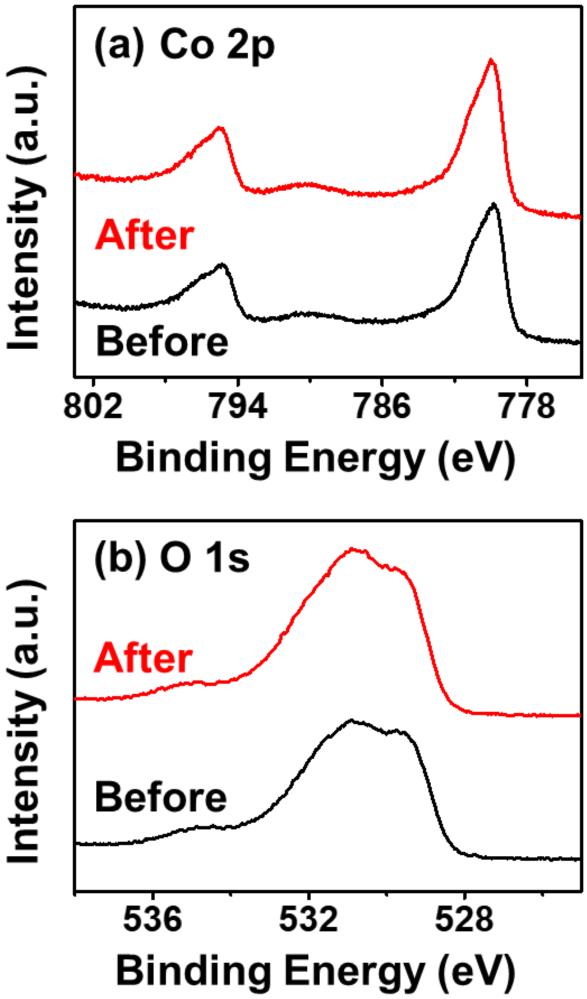

Figure S25. XPS analyses before and after the stability test of the Echem-γ-CoOOH.

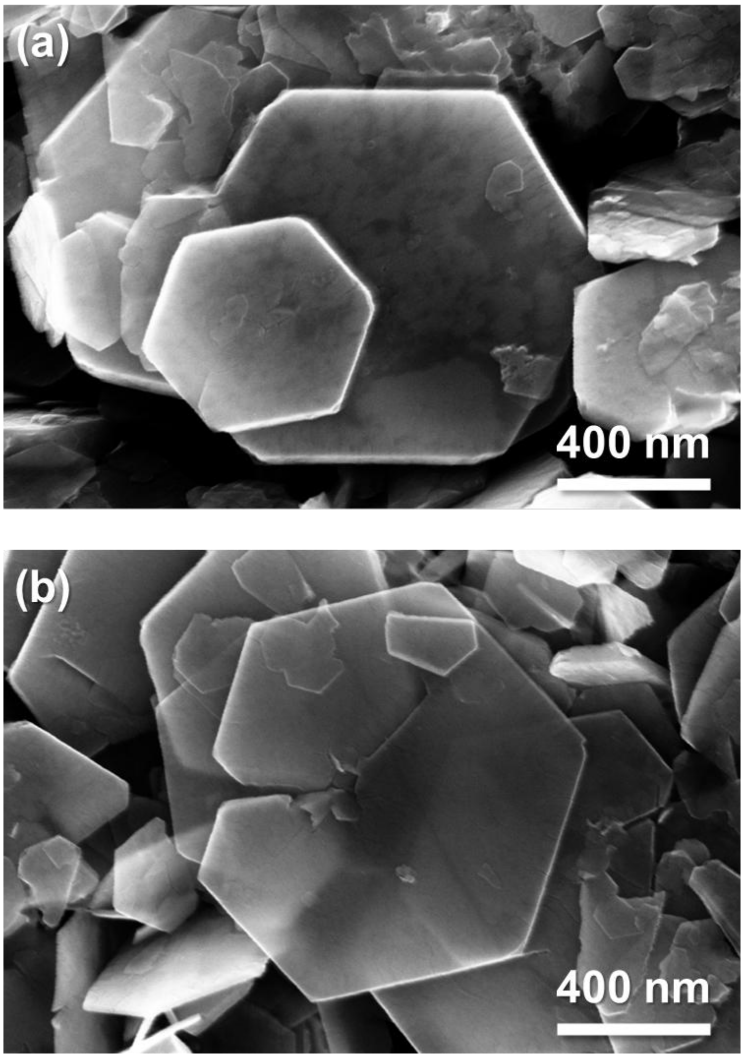

Figure S26. SEM images of Echem-γ-CoOOH (a) before and (b) after the stability test.

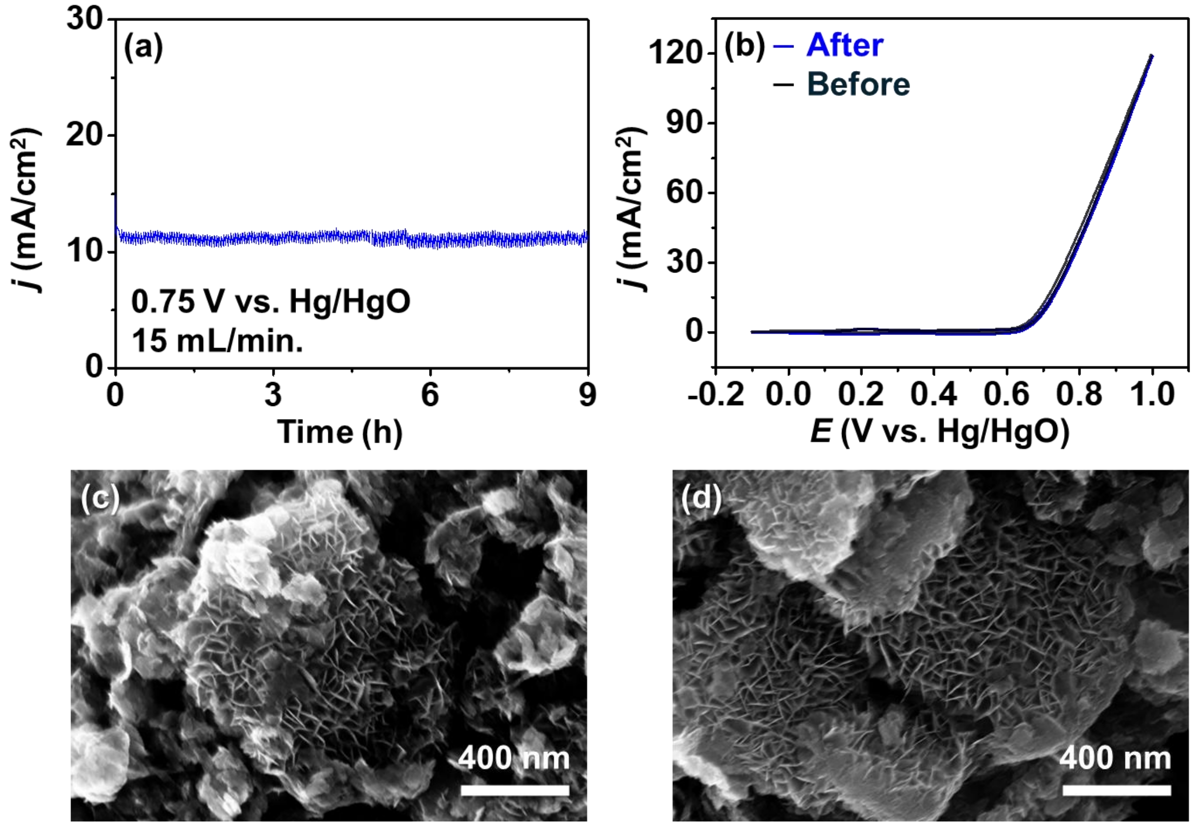

Figure S27. (a) Chronoamperometric measurement of Echem-β-CoOOH 0.75 V vs. Hg/HgO for 9 h. (b) CVs of Echem-β-CoOOH on carbon paper before and after the chronoamperometry measurement in 1 M KOH (scan rate: 10 mV s^{-1}). SEM images of Echem-β-CoOOH (c) before and (d) after the stability test.

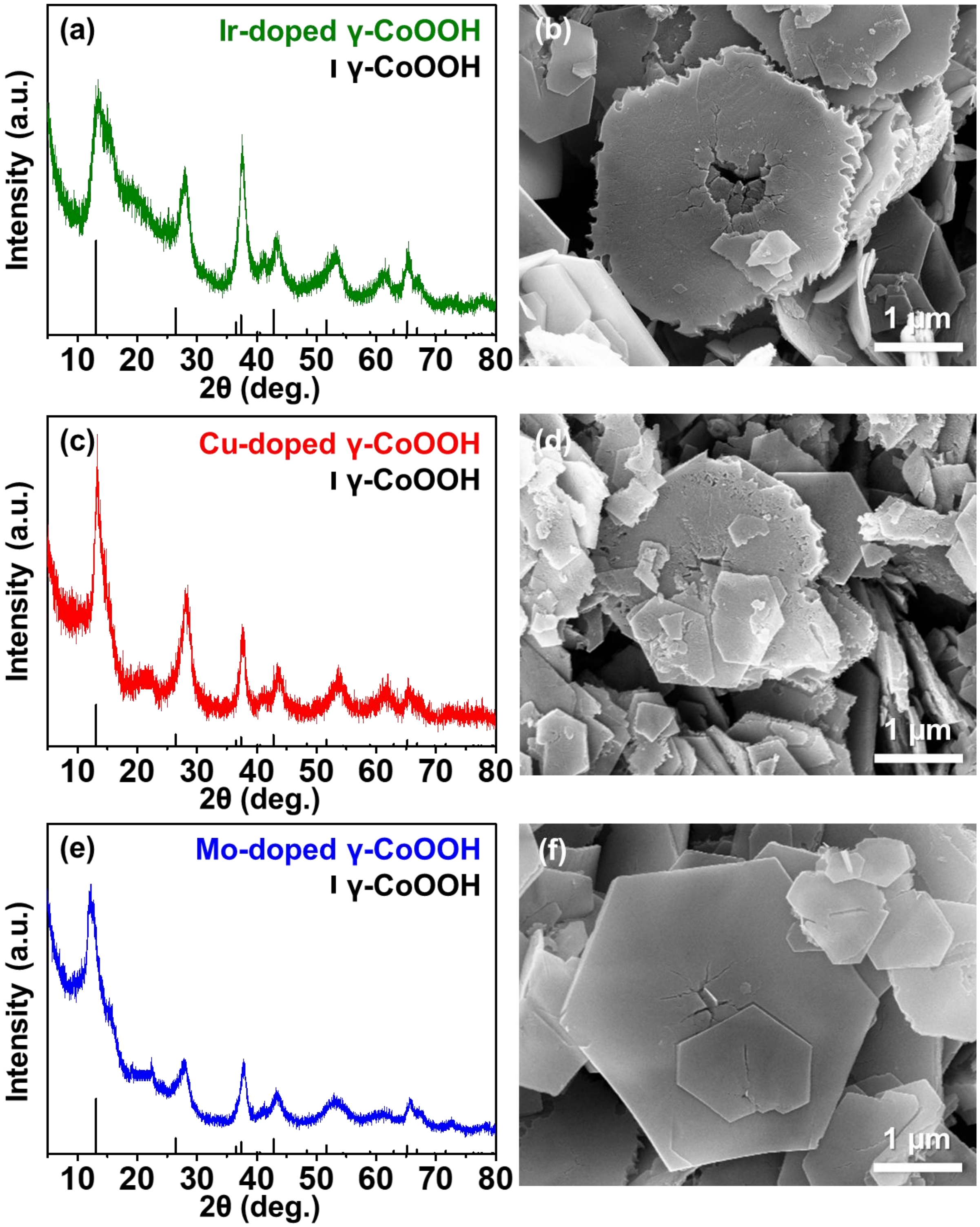

Figure S28. (a) XRD pattern and (b) SEM image of Ir-doped γ-CoOOH. (c) XRD pattern and (d) SEM image of Cu-doped γ-CoOOH. (e) XRD pattern and (f) SEM image of Mo-doped γ-CoOOH.

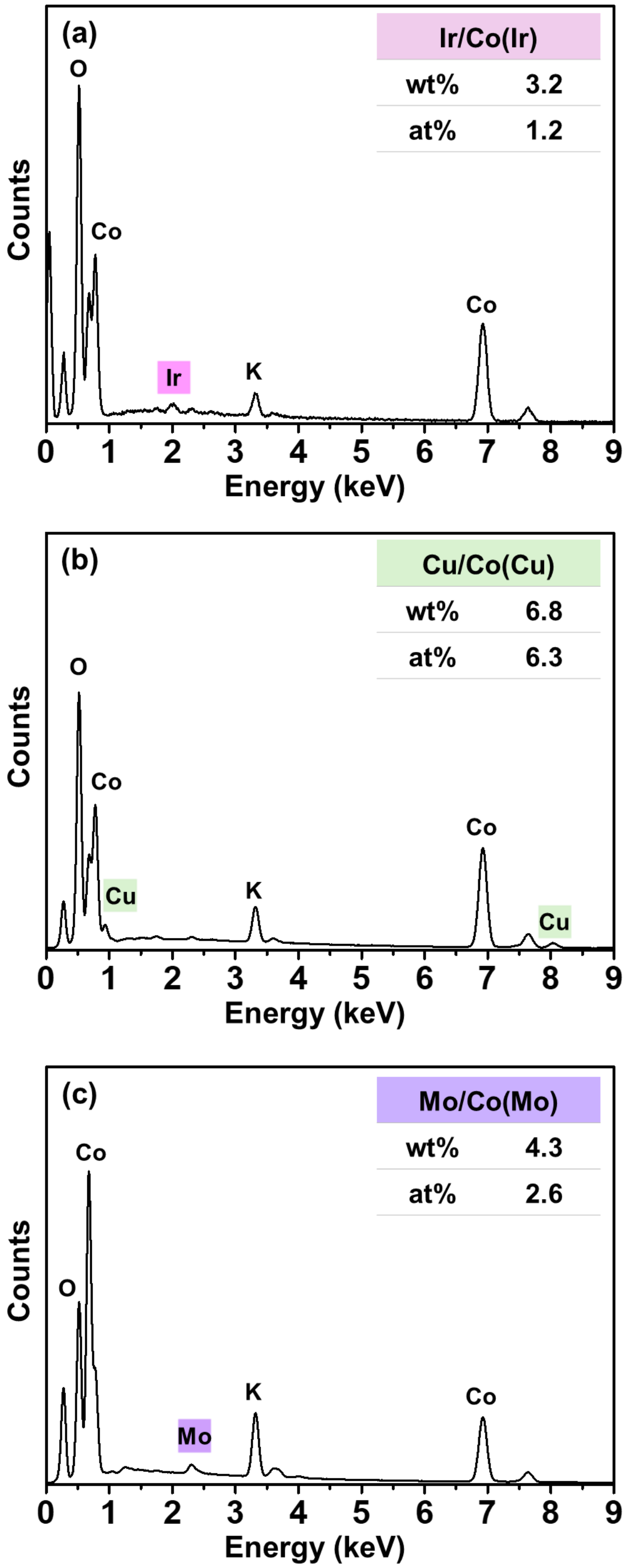

Figure S29. SEM-EDX spectra of Echem-γ-CoOOH with doped (a) Ir, (b) Cu, and (c) Mo ions. Insets show the elemental composition (wt% and at%) of each dopant relative to total metal content.

Sample		γ-CoOOH (Na⁺-intercalated)	Ir-doped γ-CoOOH	Cu-doped γ-CoOOH	δ-MnO_2 (Na⁺-intercalated)
Ratio		Na/Co	Ir/(Ir+Co)	Cu/(Cu+Co)	Na/Mn
wt%	SEM-EDS	3%	3.2%	6.8%	14%
	ICP-OES	2.5%	3.2%	8.0%	12%
at%	SEM-EDS	7.7%	1.2%	6.3%	34%
	ICP-OES	6.2%	1.2%	7.4%	25%

Figure S30. ICP-OES results for γ-CoOOH (Na^+ intercalated), Cu-doped γ-CoOOH, Ir-doped γ-CoOOH, and δ-MnO_2 (Na^+ intercalated) samples, showing the relative metal contents normalized to the Co amount.

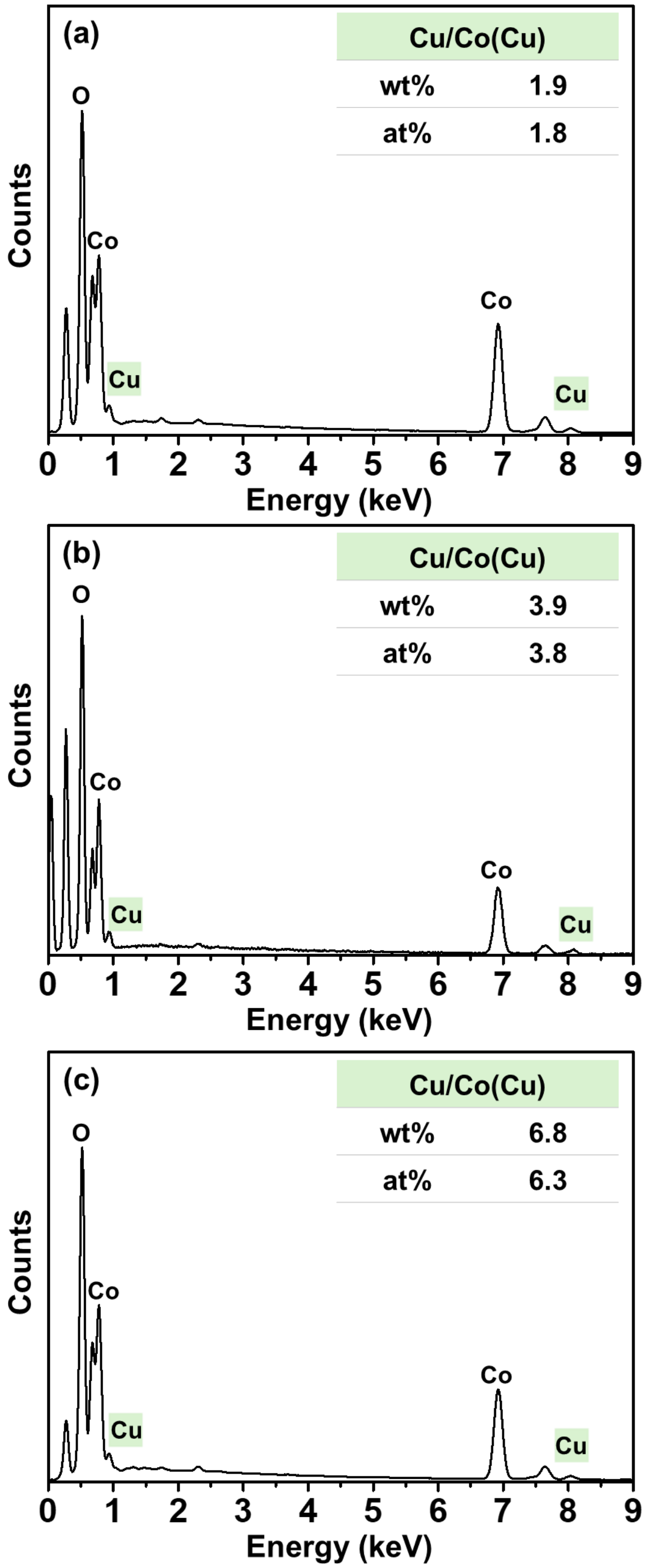

Figure S31. SEM-EDX analyses of Cu-doped γ-CoOOH samples with varying Cu dopant concentrations.

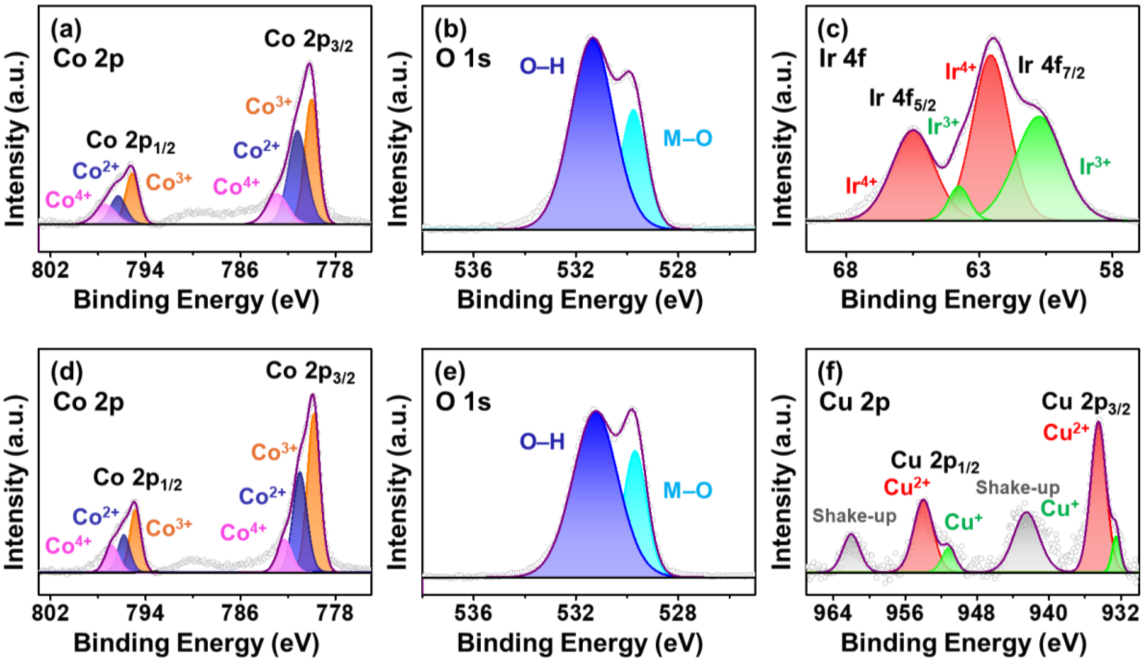

Figure S32. XPS analyses of γ-CoOOH samples doped with (a–c) Ir^{4+}, and (d–f) Cu^{2+}.

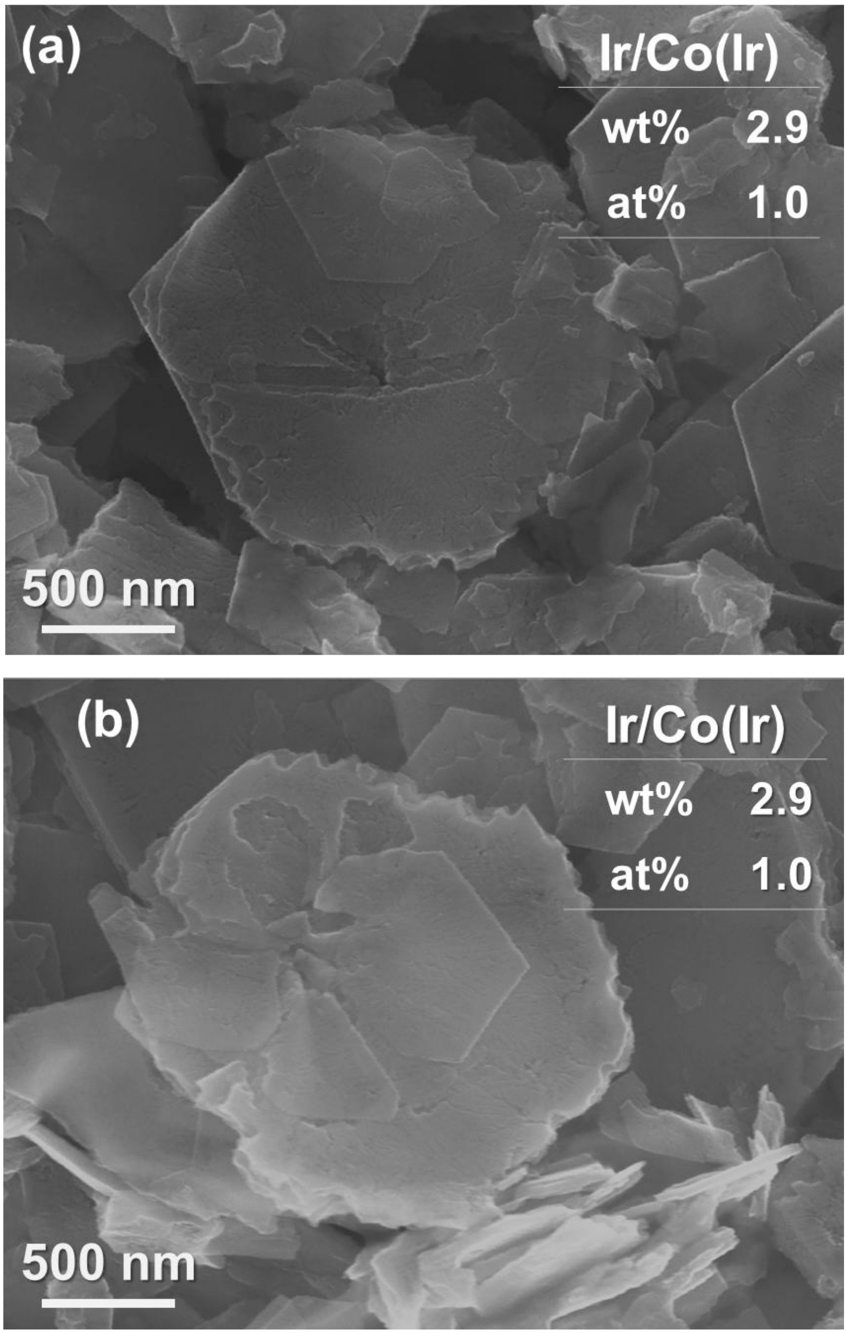

Figure S33. SEM images of Ir-doped γ-CoOOH (a) before and (b) after the electrochemical reaction. The Ir content is presented as wt% and at% relative to the total metal content (Ir and Co), quantified from SEM-EDX elemental mapping performed at the corresponding regions.
